# Unraveling the CDK9/PP2A/ERK Network in Transcriptional Pause Release and Complement Activation in KRAS‐mutant Cancers

**DOI:** 10.1002/advs.202404926

**Published:** 2024-09-10

**Authors:** Yafang Wang, Lansong Xu, Lijun Ling, Mingyue Yao, Shangxuan Shi, Chengcheng Yu, Yingnian Li, Jie Shen, Hualiang Jiang, Chengying Xie

**Affiliations:** ^1^ Shanghai Institute for Advanced Immunochemical Studies ShanghaiTech University 393 Middle Huaxia Road Shanghai 201210 P. R. China; ^2^ The First Affiliated Hospital of USTC (Anhui Provincial Hospital) Division of Life Sciences and Medicine University of Science and Technology of China Hefei Anhui 230026 P. R. China; ^3^ Lingang Laboratory Shanghai 200031 P. R. China; ^4^ School of Life Science and Technology ShanghaiTech University Shanghai 201210 P. R. China; ^5^ Drug Discovery and Development Center Shanghai Institute of Materia Medica Chinese Academy of Sciences 555 Zuchongzhi Road Shanghai 201203 P. R. China; ^6^ Department of Pharmacy The SATCM Third Grade Laboratory of Traditional Chinese Medicine Preparations Shuguang Hospital Affiliated to Shanghai University of Traditional Chinese Medicine Shanghai 201203 P. R. China

**Keywords:** KRAS, CDK9, ERK, complement activation, immunosuppression

## Abstract

Selective inhibition of the transcription elongation factor (P‐TEFb) complex represents a promising approach in cancer therapy, yet CDK9 inhibitors (CDK9i) are currently limited primarily to certain hematological malignancies. Herein, while initial responses to CDK9‐targeted therapies are observed in vitro across various KRAS‐mutant cancer types, their efficacy is far from satisfactory in nude mouse xenograft models. Mechanistically, CDK9 inhibition leads to compensatory activation of ERK‐MYC signaling, accompanied by the recovery of proto‐oncogenes, upregulation of immediate early genes (IEGs), stimulation of the complement C1r‐C3‐C3a cascade, and induction of tumor immunosuppression. The “paradoxical” regulation of PP2Ac activity involving the CDK9/Src interplay contributes to ERK phosphorylation and pause‐release of RNA polymerase II (Pol II). Co‐targeting of CDK9 and KRAS/MAPK signaling pathways eliminates ERK‐MYC activation and prevents feedback activation mediated by receptor tyrosine kinases, leading to more effective control of KRAS‐mutant cancers and overcoming KRASi resistance. Moreover, modulating the tumor microenvironment (TME) by complement system intervention enhances the response to CDK9i and potently suppresses tumor growth. Overall, the preclinical investigations establish a robust framework for conducting clinical trials employing KRASi/SOS1i/MEKi or immunomodifiers in combination with CDK9i to simultaneously target cancer cells and their crosstalk with the TME, thereby yielding improved responses in KRAS‐mutant patients.

## Graphical abstract

This study reveals the CDK9/PP2A/ERK network involved in transcriptional pause release and complement activation in KRAS‐mutant cancers, which limits the efficacy of CDK9i in vivo. The preclinical investigations establish a robust framework for conducting clinical trials employing KRASi/SOS1i/MEKi or immunomodifiers combined with CDK9i to simultaneously target cancer cells and their crosstalk with tumor microenvironment, thereby enhancing efficacy and overcoming resistance.

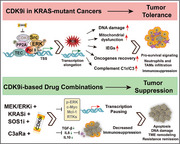

## 1 Introduction

Since the identification of oncogenes encoding protein kinases, optimism surrounding kinase inhibition as a targeted therapeutic strategy has now translated into clinical reality, including cyclin‐dependent kinases (CDKs), particularly the approved CDK4/6 inhibitors and ongoing clinical trials for other CDKs.^[^ ^1^ ^]^ Among these, CDK9, the catalytic subunit of positive transcription elongation factor b (P‐TEFb), facilitates the pause‐release of RNA polymerase II (Pol II) and reinforces the transcriptional elongation of signal‐responsive genes.^[^ ^2^ ^]^ Acute inhibition of CDK9 leads to transient suppression of transcription and preferential depletion of short‐lived proteins such as Mcl‐1^[^ ^3^ ^]^ and c‐Myc.^[^ ^4^ ^]^ Therefore, CDK9 inhibitors (CDK9i) have demonstrated therapeutic efficacy in preclinical models of Mcl‐1‐ or MYC‐driven malignancies, leading to their advancement into clinical trials. Although CDK9i have shown potent cytotoxicity against solid tumor cells in vitro studies, intrinsic resistance dominates in in vivo models. The US Food and Drug Administration (FDA) approval remains elusive due to potency and specificity concerns.^[^ ^5^ ^]^ The unsatisfactory efficacy implies an incomplete comprehension of CDK9‐interacting macromolecular complexes,^[^ ^6^ ^]^ the oncogenic pathways that CDK9i activate,^[^ ^7^ ^]^ the intricate and redundant transcription machinery,^[^ ^8^ ^]^ or other drug‐driven compensatory mechanisms. Therefore, elucidating the cellular compensation triggered by CDK9 inhibition in solid tumors may markedly potentiate the effectiveness of this potent intervention in highly refractory cancers.

Kirsten Ras (KRAS) mutation, one of the most prevalent alterations in human cancers, occurs in ≈25% of patients. These frequencies are particularly elevated in inherently lethal malignancies, such as pancreatic ductal adenocarcinoma (PDAC), colon adenocarcinoma (COAD), and non‐small cell lung cancer (NSCLC). KRAS with distinct mutations at amino acids G12, G13, and Q61 is associated with tumor aggressiveness and unfavorable patient prognosis.^[^ ^9^ ^]^ The successful development of specific inhibitors against KRAS^G12C^ variant signifies a remarkable advancement in the realm of KRAS‐targeted therapies. However, the therapeutic potential of these agents is hindered by intrinsic or rapidly emerging acquired resistance, and there is a dearth of available drugs targeting other KRAS mutants. In addition, the heterogeneity of KRAS‐mutant cancers leads to limited efficacy of KRAS inhibitors (KRASi) as monotherapy; while their combination with other targeted agents, including SHP2, EGFR, PD‐L1, CDK4/6,^[^ ^10^ ^]^ PLK1,^[^ ^11^ ^]^ and IRE1α,^[^ ^12^ ^]^ significantly enhances anti‐tumor activity and overcomes resistance. Therefore, it is imperative to delve deeper into the mechanisms underlying resistance to KRAS inhibitors and elucidate novel molecular targets that can be exploited in combinatorial treatment for the majority of patients harboring diverse KRAS mutations.

The response to KRASi is associated with on‐treatment tumor microenvironment (TME) remodeling characterized by increased effector T‐cell infiltration, anti‐tumor M1‐macrophage repolarization, and natural killer (NK) cell recruitment,^[^ ^13^ ^]^ which increase susceptibility to immunotherapies following KRAS inhibition. The synergistic benefit of combining KRASi with immune checkpoint blockade (ICB) is exclusively observed in immunogenic tumor models. However, reaching this synergy in the clinical setting has proven to be a formidable challenge, as it fails to enhance efficacy in immunogenically cold tumors, such as PDAC.^[^ ^14^ ^]^ Additionally, malignances exhibiting resistance to KRASi are frequently accompanied by an immunosuppressive TME and confer cross‐resistance to ICB treatment.^[^ ^15^ ^]^ This highlights the urgent necessity to explore the immunomodulatory characteristics of targeted agents and the therapeutic prospects of other immune components.

In this study, we employed transcriptome sequencing and interactome screens to identify the factors conferring tumor tolerance to CDK9i. We uncovered a critical mechanistic switch involving protein kinases and phosphatases that dynamically modulate Pol II‐ and ERK‐dependent transcriptional elongation. Furthermore, we demonstrated that the limited in vivo efficacy of CDK9i in KRAS‐mutant cancers can be attributed not only to the activated pro‐survival signaling in tumor cells but also to the immunosuppressive TME orchestrated by the complement system. Our findings are expected to establish an oncogene‐driven combinatorial treatment strategy to potentiate the anti‐tumor activity of both CDK9i and KRAS signaling inhibitors, while concurrently mitigating immunosuppression and overcoming drug resistance.

## 2 Results

### 2.1 CDK9i‐induced Mitochondrial Dysfunction Causes an Oxidative and Pro‐Oncogenic Environment in KRAS‐mutant Cancers

**Figure 1:**
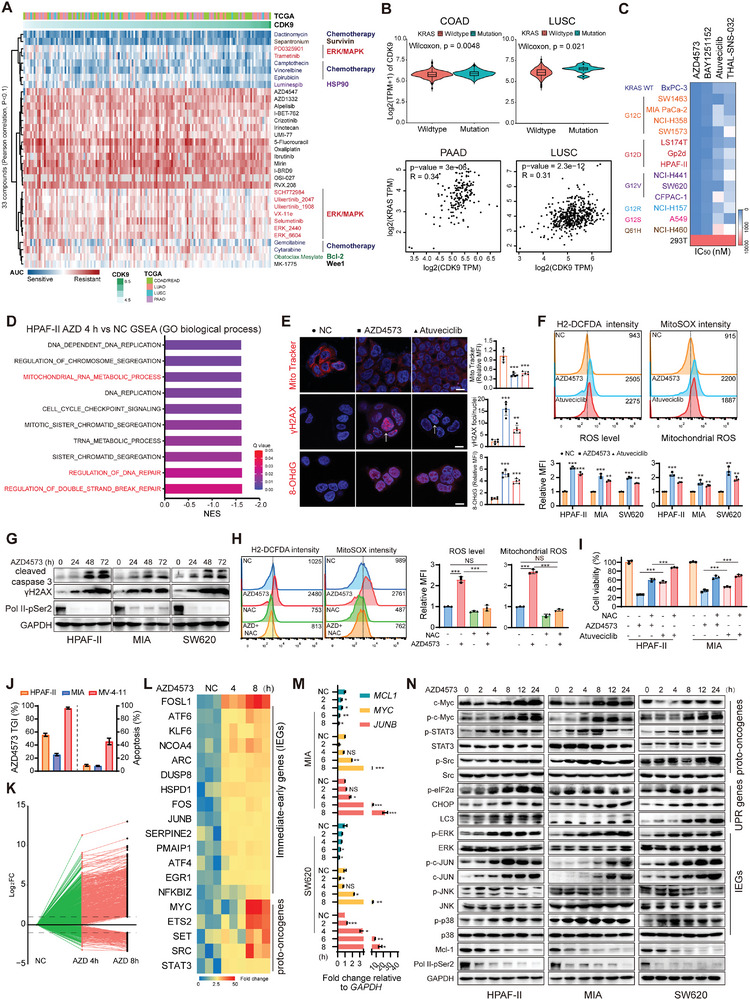
CDK9 inhibition leads to increased oxidative stress and mitochondrial dysfunction in KRAS‐mutant cancer cells. A) Hierarchical clustering of drug AUC values from COAD, READ, PAAD, LUAD, and LUSC cancer cell lines. CDK9 expression levels are shown at the top of the heatmap. Only drugs with Pearson correlation p values < 0.1 were included. A low AUC value (blue) indicates sensitivity to drug treatment. B) Quantitative CDK9 levels in samples from COAD (KRAS WT: 284 cases; mutant: 174 cases) and LUSC (KRAS WT: 490 cases; mutant: 8 cases) patients (top). Spearman's correlation analysis of CDK9 and KRAS expression levels in PAAD and LUSC samples were performed on the GEPIA database (down). C) Heatmap showing the IC_50_ values of the indicated CDK9i in diverse cell lines harboring WT or mutant KRAS. D) Bar plots showing GSEA of GO biological processes in HPAF‐II cells treated with 40 nM AZD4573 for 4 h. Normalized enrichment score (NES) and Q‐values are shown. E) Immunofluorescence (IF) staining of MitoTracker, γH2AX, and 8‐OHdG in HPAF‐II cells treated with 40 nm AZD4573 or 1 µm Atuveciclib for 24 h. Scale bar: 10 µm. F) H_2_‐DCFDA and MitoSOX distribution were measured by flow cytometry in HPAF‐II, MIA, and SW620 cells treated with 40 nm AZD4573 or 1 µm Atuveciclib for 8 h. G) Western blot analysis of cleaved caspase‐3, γH2AX and Pol II‐pSer2 levels in HPAF‐II, MIA and SW620 cells treated with 40 nm AZD4573 for 24, 48 and 72 h. H) Preincubation of MIA cells with the ROS inhibitor NAC (5 mM) for 1 h blocked the generation of intracellular and mitochondrial ROS induced by AZD4573. I) Viability of HPAF‐II and MIA cells following CDK9i treatment with or without 5 mM NAC (mean ± SD, *n =* 3 biological replicates). J) TGI rates of 15 mg kg^−1^ AZD4573, twice daily with a 2‐h split dose (BID q2h), on 2 days on/5 days off schedule in the xenograft models of HPAF‐II, MIA, and MV‐4‐11 (*n =* 3, left). Cell apoptosis of these cell lines after treatment with 40 nm AZD4573 for 8 h in vitro was analyzed by flow cytometry following Annexin V/PI double staining (right). K) Dynamic expression of genes that were significantly deregulated (|fold change (FC)|>2, FDR<0.05) after 8 h AZD4573 (40 nm) treatment. The black dotted lines represent a two‐fold change. L) Heatmap showing selected upregulated genes involved in IEGs and proto‐oncogenes after CDK9 inhibition in HPAF‐II cells. M) RT‐qPCR was used to determine the mRNA levels of *MCL1*, *MYC* and *JUNB* in MIA and SW620 cells after treatment with 40 nm AZD4573 for 2, 4, 6, 8 h. N) Effect of AZD4573 (40 nm) on the time‐course expression of proto‐oncogenes, UPR genes and IEGs in HPAF‐II, MIA and SW620 cells. Data are presented as mean ± SD (*n =* 3). *p < 0.05, **p < 0.01, ***p < 0.001 by unpaired Student's *t* test.

To comprehensively investigate the therapeutic potential of targeting CDK9 in solid tumors, we analyzed the drug screening data from the Cancer Therapeutics Response Portal (CTRP) to identify associations between CDK9 expression and drug sensitivity. We filtered drugs through area under the curve (AUC) values that exhibited significant correlations with CDK9 expression in COAD, rectum adenocarcinoma (READ), lung adenocarcinoma (LUAD), lung squamous cell carcinoma (LUSC), and pancreatic adenocarcinoma (PAAD) cancer cell lines (**Figure** **1**A). The top candidates included drugs targeting HSP90 (Luminespib), MEK (PD0325901), ERK (ERK_6604 and SCH772984), c‐Met (Crizotinib) and Bcl‐2 (UMI‐77). CDK9 levels exhibited inverse correlations with the AUCs of these drugs (Figure S1A, Supporting Information), suggesting that CDK9 might be associated with the sensitivity to the drugs of these targets. No discernible variation in sensitivity was observed for FGFR, NRTK, or BTK inhibitors (Figure S1B, Supporting Information). Generally, the data suggested a striking correlation between CDK9 dependency and ERK/MAPK signaling. Moreover, based on the TCGA database, CDK9 expression was significantly higher in COAD or LUSC KRAS‐mutant patients than in wild‐type (WT) patients (Figure 1B). Correlation analyses confirmed a positive association between the expressions of CDK9 and KRAS/MAPK1 in PAAD, LUSC, and COAD patients (Figure 1B; Figure S1C, Supporting Information). The expression of CDK9 was also upregulated in cell lines with different types of KRAS mutations compared to KRAS WT cell BxPC‐3 and normal epithelial cell 293T (Figure S1D, Supporting Information). Then, we evaluated the anti‐proliferative effects of CDK9i, including AZD4573, BAY1251152, Atuveciclib, and THAL‐SNS‐032 on these cells with diverse KRAS status. Cell lines harboring either WT or mutated KRAS exhibited similar sensitivity to CDK9i in vitro with IC_50_ values mostly <1 µm. In contrast, normal epithelial cells 293T were insensitivity to these inhibitors (Figure 1C). These findings suggest that targeting KRAS‐driven cancers via CDK9i in a cell‐autonomous manner is a pharmacologic approach.

We next performed genome‐wide transcriptome analyses using a pancreatic cancer cell line HPAF‐II (KRAS^G12D^) treated with or without AZD4573 to elucidate the mechanism underlying CDK9i‐induced cell death. Gene set enrichment analysis (GSEA) of gene ontology (GO) biological processes and HALLMARK gene sets revealed that the most significantly negatively enriched pathways included DNA replication, mitochondrial RNA metabolic processes, and DNA repair (Figure 1D; Figure S1E, Supporting Information). The gene signature of mitochondrial RNA metabolic processes revealed a robust reduction following AZD4573 treatment (Figure S1F, Supporting Information), with some key regulators of mitochondrial function further confirmed by RT‐qPCR analysis (Figure S1G, Supporting Information). Cellular immunofluorescence showed that CDK9i induced a notable decrease in the mitochondrial membrane potential indicated by JC‐1 staining, concomitant with mitochondrial swelling and oxidative DNA damage, as evidenced by increased γH2AX and 8‐hydroxy‐2‐deoxyguanosine (8‐OHdG) signals (Figure 1E; Figure S1H, Supporting Information). In addition, mitochondrial respiration using the Seahorse Mito Stress assay showed that major parameters of the oxygen consumption rate (OCR), including basal respiration, maximal respiration, and ATP production, were all reduced in HPAF‐II cells following AZD4573 treatment (Figures S1I,J, Supporting Information), indicating that mitochondrial function was impaired. Moreover, CDK9i repressed the expression of antioxidant proteins HIF‐1α and NRF2 (Figure S1K, Supporting Information) while triggering the generation of intracellular reactive oxygen species (ROS) and mitochondrial ROS (mtROS) (Figure 1F), ultimately leading to a significant increase in γH2AX and cleaved caspase‐3 upon prolonged treatment (Figure 1G). When excessive ROS were effectively counteracted by the antioxidant treatment with N‐acetylcysteine (NAC) (Figure 1H), CDK9i‐induced cell death was partially alleviated and γH2AX level was reduced (Figure 1I; Figure S1L, Supporting Information). Taken together, these findings reveal that CDK9i inhibit cell proliferation in vitro, partially through the induction of ROS accumulation, mitochondrial dysfunction, and DNA damage.

However, the response to CDK9i in nude mouse tumor models has not been satisfactory. Both KRAS‐mutant HPAF‐II and MIA PaCa‐2 (MIA) cell xenografts were refractory to AZD4573, with average tumor growth inhibition (TGI) rates of 58% and 27%, respectively, compared to 97% in an acute myelocytic leukemia (AML) cell xenograft model (MV‐4‐11) under the same treatment condition (Figure 1J). Consistent with this finding, AZD4573 induced rapid apoptosis in AML cells, with over 40% apoptotic cell death within 8 h, demonstrating greater potency than that observed in HPAF‐II and MIA cells (Figure 1J). To elucidate the underlying mechanism, we dug into the RNA‐seq data using HPAF‐II cells treated with AZD4573 for 4 or 8 h (Figure S1M, Supporting Information). Short‐term CDK9 inhibition (4 h) primarily resulted in gene downregulation (700 down vs 666 up). In contrast to this initial response, the expression of massive genes was significantly upregulated at 8 h (676 up versus 320 down, Figure S1M,N, Supporting Information), with certain genes exhibiting recovery following downregulation at 4 h AZD4573 treatment (Figure 1K). Among these, 521 genes were simultaneously differentially expressed (Figure S1O, Supporting Information). GO analysis of the commonly downregulated genes revealed significant enrichment in DNA binding and regulation of transcription. While the commonly induced genes were associated with enrichment in receptor binding and immune response (Figure S1P, Supporting Information). The reversal of gene silencing after CDK9 inhibition was unexpected but partially aligned with the findings of Hanghang Zhang et al.,^[^ ^16^ ^]^ indicating that long‐term CDK9 inhibition has a bimodal effect with one subset of genes being initially downregulated and another subset being initially upregulated, resulting in remarkably induced transcription profiles.

The efficacy of protein kinase inhibitors is frequently hindered by the emergence of adaptive resistance, characterized by kinome reprogramming and feedback compensation. Specifically, a large panel of immediate‐early genes (IEGs) such as *FOSL1*, *ATF6*, and *JUNB*, as well as pro‐survival genes, such as *MYC*, *ETS2*, and *SRC* were strongly induced after CDK9 inhibition (Figure 1L). A time‐dependent qPCR analysis showed that, unlike the continuously‐induced *JUNB*, *MYC* was promptly inhibited as early as 2 h but recovered 8 h following a single exposure (Figure 1M). The expression of *MCL1*, a well‐known P‐TEFb target, was consistently suppressed, suggesting that the induced or recovered gene expressions were directly associated with on‐target CDK9 inhibition (Figure 1M). We further verified the consistent change of these genes at protein levels in CDK9i‐treated cells (Figure 1N). AZD4573 treatment caused unfolded protein response (UPR)‐associated autophagy, as evidenced by the early induction of phosphorylated eIF2α, CHOP, and phosphatidyl‐ethanolamine‐conjugated LC3 (LC3‐II). We also observed a rapid and potent activation of ROS downstream signaling, including the phosphorylation of c‐Jun, ERK1/2, and p38 MAPK (Figure 1N). Similar upregulations were also confirmed by using another CDK9i Atuveciclib (Figure S1Q, Supporting Information). Taken together, these findings indicate that mitochondrial defects induced by CDK9 inhibition not only trigger ROS generation and DNA damage but also establish an oxidative and pro‐survival/carcinogenic microenvironment, potentially leading to an insufficient in vivo response to CDK9i in KRAS‐mutant cancers. Importantly, although excessive ROS are known to activate MAPK signaling under cellular stress conditions,^[^ ^17^ ^]^ ERK activation following long‐term CDK9i treatment remained unaffected by NAC, suggesting additional mechanisms driving ERK phosphorylation (Figure S1L, Supporting Information).

### 2.2 Systematic Determination of the CDK9 Interactome Reveals Novel Compensations for Transcription Elongation

**Figure 2:**
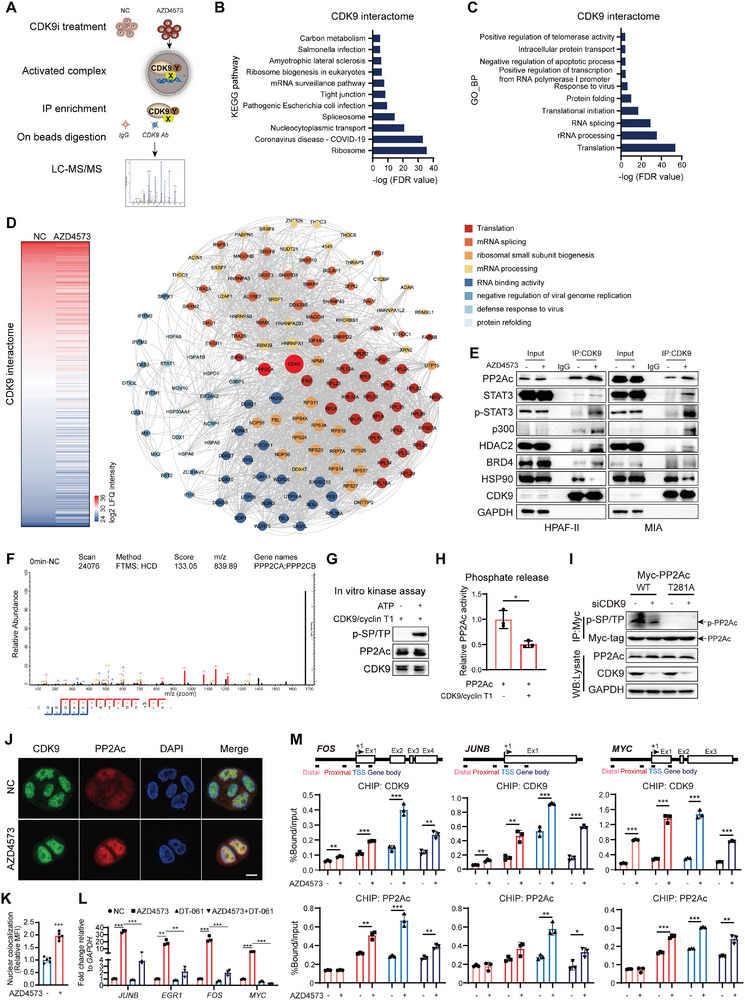
The CDK9 interactome potentiates the transcriptional elongation of IEGs and oncogenes after CDK9i treatment. A) A diagram showing the flow chart of label‐free quantitative proteomics analysis by LC‐MS/MS using CDK9‐immunoprecipitates from HPAF‐II control‐ or AZD4573‐treated (40 nm, 8 h) cells. KEGG B) and GO‐BP analyses C) of the proteins identified with high confidence in CDK9 interactome using the DAVID database. D) The deregulated CDK9‐interacting proteins in AZD4573‐treated cells were subjected to protein–protein interaction (PPI) analysis with STRING. The warm‐colored panel shows the significant functional enrichment of the upregulated PPI network; the cold‐colored panel shows the downregulated ones. E) Co‐IP of CDK9 with the indicated proteins in HPAF‐II and MIA cells treated with or without 40 nm AZD4573 for 8 h. F) Secondary protein profile showing the phosphorylation site on the PPP2CA/PPP2CB peptide. G) Recombinant CDK9/cyclin T1 was incubated with 6His‐PP2Ac‐WT protein expressed in *E. coli* and purified using Ni‐NTA agarose resin. Phosphorylation was detected with an anti‐p‐SP/TP antibody. H) PP2Ac activity was analyzed by an in vitro phosphate release assay. Endogenous PP2Ac was immunoprecipitated and subjected to the CDK9/cyclin T1 complex using a threonine‐phosphopeptide substrate. I) MIA cells expressing Myc‐PP2Ac‐WT or ‐T281A were treated with siNC or siCDK9. The phosphorylation of immunoprecipitated Myc‐PP2Ac (WT and T281A) was probed with an anti‐p‐SP/TP antibody. Representative IF images (*n =* 5 images in each group) J) and quantitative analysis K) showing the co‐localization of PP2Ac and CDK9 in MIA cells upon AZD4573 treatment (40 nm, 8 h). L) RT‐qPCR analysis of IEGs mRNA expression in HPAF‐II cells after treatment with 40 nm AZD4573 and 10 µm DT‐061 for 8 h. M) Distribution of CDK9 and PP2Ac on the chromatin sites encoding *FOS*, *JUNB* and *MYC* genes with or without AZD4573 (40 nm, 8 h) treatment. CHIP‐qPCR assays were performed with an anti‐CDK9 antibody (top) or an anti‐PP2Ac antibody (bottom) using chromatin prepared from HPAF‐II cells. The densities of CDK9 and PP2Ac are presented as a percentage of the input. Data are presented as mean ± SD (*n =* 3). *p < 0.05, **p < 0.01, ***p < 0.001 by unpaired Student's *t* test.

We uncovered that although the phosphorylation of Ser2 on the C‐terminal domain (CTD) of Pol II is inhibited upon CDK9i treatment, gene transcription is not completely stalled. Given the frequent collaboration of CDK9 with other factors, we posited that the inhibition of CDK9 activity likely alters its interactome, potentially revealing the compensatory interactions involved in transcription elongation. To broadly characterize how CDK9 partners change, we subjected control‐ and AZD4573‐treated HPAF‐II cells to immunoprecipitation (IP) enrichment coupled with label‐free relative quantitative proteomics (LFQ) using LC‐MS/MS analysis (**Figure** **2**A). Consequently, we identified a total of 1235 confidently detected proteins composing the CDK9 interactome across duplicate experiments. KEGG and GO biological process analyses of the identified proteins revealed significant enrichment of ribosome‐related processes, spliceosome assembly, and translation (Figure 2B,C). We further selected the high‐scoring (≥24) proteins and analyzed those with LFQ intensities ≥ 2‐fold differences in the STRING database to construct cluster networks. The basal CDK9 complexes were enriched in RNA binding activity and protein refolding (highlighted in blue bands, Figure 2D); while the AZD4573‐upregulated subnetworks were enriched in translation and mRNA splicing (highlighted in red bands, Figure 2D). Additionally, AZD4573 enhanced the interaction of CDK9 with chromatin‐modifying complexes such as bromodomain‐containing protein 4 (BRD4), E1A binding protein p300 (p300), signal transducer and activator of transcription 3 (STAT3), and histone deacetylases (HDACs), as well as certain protein kinases/phosphatases, but reduced binding to heat shock proteins (Figure 2E; Table S5, Supporting Information). Collectively, these results indicate that CDK9 inhibition induces significant dynamic changes in the CDK9 interactome, which sparkle with crucial roles in chromosome remodeling, transcription elongation, and translation.

We identified six peptides associated with serine/threonine phosphatases within the CDK9 interactome: PPP2CA, PPP2CB, PPP2R1A, PPP2R2A, PPP1CA and PPP1CB (Figure S2A, Supporting Information). Although it has been reported that the loss of function of the INTS6/PP2A axis drives escape from CDK9i‐induced pausing by controlling the turnover of phosphorylated Pol II C‐terminal domain (CTD) and DRB sensitivity‐inducing factor (DSIF),^[^ ^18^ ^]^ the mechanism by which PP2A is suppressed under normal physiological conditions remains elusive. Additionally, CDK9 can directly inactivate protein phosphatase PP1γ through inhibitory phosphorylation.^[^ ^19^ ^]^ Based on these, we postulated that CDK9 might also negatively regulate PP2A activity. The LC‐MS/MS result revealed a phosphorylation site [threonine‐281 (T281)] on the peptide of PP2A catalytic subunit alpha (PP2Ac), exclusively in nontreated HPAF‐II cells (Figure 2F). Multiple‐sequence alignment revealed high conservation of this residue across species, suggesting its potentially important role in most organisms (Figure S2B, Supporting Information). CDK9i treatment reduced the total serine/threonine phosphorylation (p‐SP/TP) of PP2Ac (Figure S2C, Supporting Information). We then performed an in vitro kinase assay by incubating recombinant 6His‐PP2Ac protein, which was expressed in *E. coli* and purified using metal chelate affinity chromatography on Ni‐NTA agarose resin, with recombinant CDK9/cyclin T1 complex. The p‐SP/TP signal was detected in 6His‐PP2Ac under the presence of ATP (Figure 2G), suggesting that CDK9 directly phosphorylates PP2Ac on threonine residues. To assess the impact of CDK9‐dependent phosphorylation on PP2Ac activity, an in vitro phosphate release assay was performed and indicated that CDK9/cyclin T1 reduced the phosphatase activity of PP2Ac by ≈50% (Figure 2H). To further verify this in KRAS‐mutant cells, immunoprecipitation of PP2Ac from MIA cell lysates also demonstrated an endogenous p‐SP/TP‐modified PP2Ac, which was greatly attenuated in both CDK9‐knockdown (Figure 2I) and CDK9‐inhibited cells (Figure S2D, Supporting Information). Thr‐to‐Ala substitution at this site (T281A) effectively abolished most p‐SP/TP on PP2Ac (Figure 2I; Figure S2D, Supporting Information). Moreover, cellular immunofluorescence showed enhanced nuclear colocalization of CDK9 and PP2Ac in MIA cells treated with AZD4573 (Figure 2J,K). In conclusion, CDK9 phosphorylates PP2Ac at the conserved T281 residue and restricts its phosphatase activity.

It has been reported that PP2Ac colocalizes with Pol II and mediates its dephosphorylation across the genome, suggesting a potential role of PP2Ac recruitment on transcriptional output.^[^ ^20^ ^]^ DT‐061, an allosteric agonist of PP2A, effectively reversed the upregulation of IEGs and *MYC* induced by CDK9i (Figure 2L), indicating that PP2Ac with a low phosphatase activity was involved in the gene induction. We next analyzed the abundance of CDK9 and PP2Ac at the loci of these genes. Chromatin immunoprecipitation‐qPCR (ChIP‐qPCR) assays revealed a pronounced increase in the abundance of CDK9 and PP2Ac along the gene body and transcription start sites (TSS) of *JUNB*, *FOS*, and *MYC* upon AZD4573 treatment (Figure 2M), suggesting a productive elongation. Collectively, these data suggest that CDK9 inhibition not only facilitates the release of PP2Ac activity but may also promote its recruitment to other regulatory modules at the TSS to reduce Pol II susceptibility to CDK9i‐induced pausing, ultimately resulting in a recovered or increased rate of transcription elongation.

### 2.3 CDK9/Src Interplay Mediates PP2Ac Inactivation and ERK Phosphorylation

**Figure 3:**
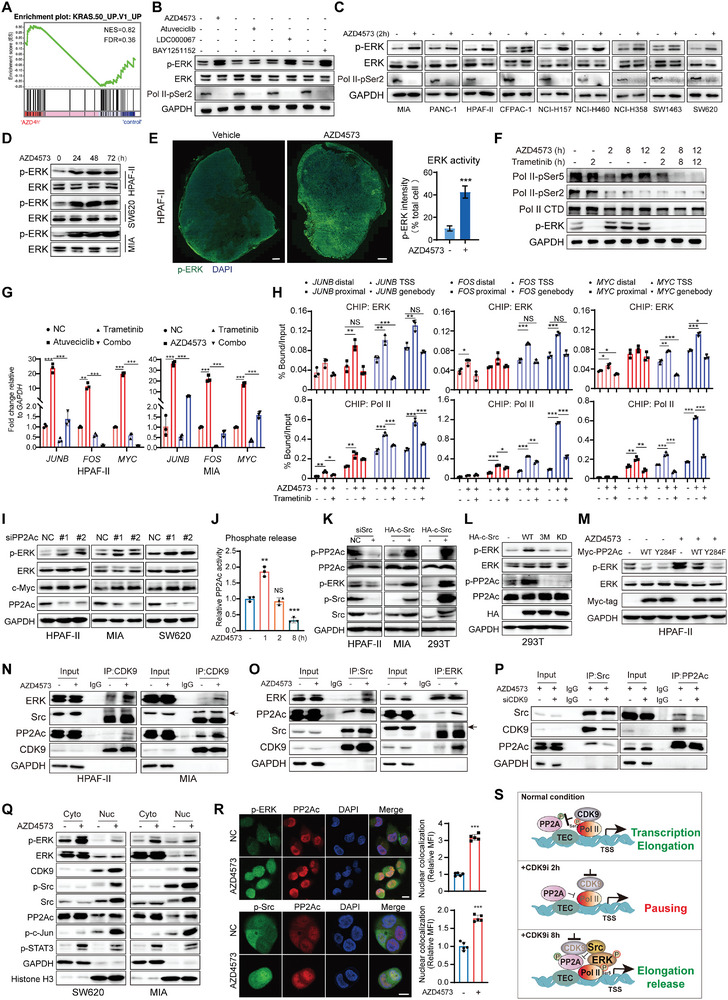
The Src/PP2Ac/ERK complex recruited to chromatin counteracts CDK9 inhibition and resumes transcription elongation. A) GSEA plots of representative gene sets associated with KRAS signaling after AZD4573 treatment for 4 h. The NES and false discovery rate (FDR) are shown. B) ERK activation in HPAF‐II cells treated with different CDK9i (40 nm AZD4573, 1 µm Atuveciclib, 20 µm LDC000067, and 40 nm Bay1251152) for 4 h. C) ERK activation in different KRAS‐mutant cell lines treated with 40 nm AZD4573 for 2 h. D) Long‐term CDK9 inhibition (40 nm AZD4573 for 24, 48, and 72 h) triggered the activation of ERK in HPAF‐II, MIA, and SW620 cells. Cells were treated for the indicated intervals and assessed for levels of p‐ERK and total ERK. E) Immunofluorescence analysis of p‐ERK in the tumor areas of HPAF‐II cell xenografts treated with vehicle or 15 mg kg^−1^ AZD4573 (BID q2h, 2 days on/5 days off, 21 days) (scale bar: 1 mm). Volumetric quantification of the overall p‐ERK immunoreactivity in tumor tissues are shown. F) Immunoblotting for Pol II p‐Ser5 and p‐Ser2 in HPAF‐II cells treated with AZD4573 (40 nm), Trametinib (100 nm), alone or in combination for indicated times. G) Bar plots showing the expression levels of IEGs in HPAF‐II and MIA cells treated with 1 µm Atuveciclib or 40 nm AZD4573 and 100 nm Trametinib for 8 h. H) CHIP‐qPCR assays were performed with anti‐ERK or anti‐Pol II antibodies using chromatin prepared from HPAF‐II cells that were treated with 40 nm AZD4573 and 100 nm Trametinib for 8 h. I) Immunoblotting showing ERK phosphorylation and c‐Myc upregulation in cells after PP2Ac knockdown. J) After treatment with AZD4573 (40 nm) for 1, 2, or 8 h in HPAF‐II cells, endogenous PP2Ac was immunoprecipitated and subjected to an in vitro phosphatase assay using a threonine‐phosphopeptide as a substrate. K) Immunoblotting showing PP2Ac and ERK phosphorylation in Src‐knockdown or ‐overexpressing cells. L) After 24 h of transfection with HA‐Src‐WT, Src‐3 M or Src‐KD, ERK, and PP2Ac phosphorylation in 293T cells expressing Src variants were determined by western blot analysis. M) HPAF‐II cells were transfected with Myc‐PP2Ac WT or Y284F mutant and treated with or without AZD4573 for another 8 h. p‐ERK were analyzed by western blot analysis. N) Co‐IP assays of exogenous CDK9 with Src, PP2Ac, or ERK in HPAF‐II and MIA cells exposed to AZD4573 (40 nm) for 8 h. O) IP‐western blot assays of untreated or AZD4573‐treated HPAF‐II cells immunoprecipitated with IgG, anti‐Src or anti‐ERK as indicated. P) IP‐Western blot assays of siNC‐ or siCDK9‐treated HPAF‐II cells immunoprecipitated with IgG, anti‐Src, or anti‐PP2Ac as indicated. Q) After treatment with AZD4573 (40 nm) for 8 h, chromatin, and cytoplasmic proteins were separated by NE‐PER extraction reagents and detected by western blotting. R) Immunofluorescence confocal microscopy showing co‐localization of endogenous p‐ERK/p‐Src and PP2Ac in MIA cells treated with 40 nm AZD4573 for 8 h. DAPI‐stained nuclei are shown in blue. Scale bar = 10 µm. Data are presented as mean ± SD (*n =* 3). *p < 0.05, **p < 0.01, ***p < 0.001 by unpaired Student's *t* test. NS indicates non‐significant. S) A transcription elongation network comprising CDK9, PP2A, Src, ERK, and other transcription elongation complexes (TEC). At or near the TSS, short‐term treatment with CDK9i releases PP2A due to a decrease in CDK9 activity and triggers Pol II pausing. After prolonged treatment, CDK9 recruits Src to mediate the suppression of PP2A and phosphorylation of ERK and Pol II CTD at Ser5, resuming transcription elongation.

We next tried to uncover the potential regulatory modules and detail the mechanism of PP2Ac inactivation after long‐term CDK9i inhibition. It was worth noting that CDK9i robustly and consistently induced the activation of ERK, an important target of PP2Ac,^[^ ^21^ ^]^ in KRAS‐mutant cancer cells (Figure 1N; Figure S1Q, Supporting Information). GESA of oncogene signatures revealed that KRAS signaling was enriched in HPAF‐II cells treated with AZD4573 (**Figure** **3**A). The induction of ERK phosphorylation by other CDK9i was further validated in all of these tested KRAS‐mutant cell lines (Figure 3B,C). Prolonged CDK9i treatment exhibited a sustained ERK activation lasting for 48 and 72 h (Figure 3D). Subsequently, tissue immunofluorescence imaging demonstrated a prominent phosphorylated‐ERK (p‐ERK) signal at the HPAF‐II tumor sites following AZD4573 administration (Figure 3E). Previous studies have reported that ERK promotes IEGs expression by phosphorylating negative elongation factor (NELF)‐A^[^ ^22^ ^]^ and Pol II CTD at Ser5^[^ ^23^ ^]^ to resume elongation. We proposed that ERK activation might be essential for gene recovery or induction following CDK9i treatment. The CDK9i‐mediated loss of phosphorylation of Pol II CTD at Ser5 was restored after prolonged AZD4573 treatment, which was alleviated by co‐treatment with Trametinib (MEK/ERK inhibitor) (Figure 3F). Moreover, concurrent repression of CDK9 and ERK signaling significantly attenuated the mRNA transcription for *JUNB*, *FOS*, and *MYC* in HPAF‐II and MIA cells (Figure 3G). Knockdown of ERK1/2 prior to CDK9i treatment also markedly reduced gene induction (Figure S3A, Supporting Information). By analyzing CHIP‐seq data from the online dataset (GSE85089),^[^ ^24^ ^]^ we found that Trametinib abrogated the EGF‐dependent recruitment of Pol II to IEGs (*FOS* and *JUNB*) and oncogenic *MYC* at their TSS in A549 cells (Figure S3B, Supporting Information). We then investigated the association of ERK with the loci of these three genes. CDK9 inhibition enhanced the accumulation of both Pol II and ERK at TSS and in gene body regions, which were significantly reduced by Trametinib treatment (Figure 3H), confirming the ERK‐induced transition of paused Pol II to productive elongation in KRAS‐mutant cells. Notably, akin to the recovery of c‐Myc protein levels observed at 6–8 h post AZD4573 treatment, reactivation of p‐c‐Myc occurred concomitantly (Figure S3C, Supporting Information), possibly mediated by p‐ERK, thereby enhancing the stability of c‐Myc. Hence, p‐ERK induced by CDK9i restored c‐Myc both in terms of gene transcription and posttranslational modification. We then examined these compensatory pathways upon CDK9 inhibition in KRAS WT cells. Interestingly, despite inducing p‐ERK in BxPC‐3 cells, both AZD4573 and Atuveciclib were unable to restore c‐Myc expression, potentially due to the lack of dependence on ERK for c‐Myc regulation in KRAS WT cell lines.^[^ ^25^ ^]^ Moreover, in 293T cells with low RAS/MAPK signaling activation, CDK9i treatment elicited a negligible effect on ERK and a sustained reduction in c‐Myc expression (Figure S3D, Supporting Information). We, therefore, speculated that KRAS WT cells, which have a lower expression level of CDK9 compared to KRAS‐mutant cells, exhibit similar sensitivity to CDK9i due to the lack of ERK‐MYC feedback activation. These findings suggest that KRAS status affects the response to CDK9i, providing a rational for investigating combination strategies to enhance the efficacy of CDK9i, particularly in tumors harboring KRAS mutations.

We further determined the role of PP2Ac in CDK9i‐induced ERK activation. Knockdown of PP2Ac by siRNAs in KRAS‐mutant cells increased the levels of p‐ERK and c‐Myc, similar to the response induced by CDK9i (Figure 3I; Figure S3E, Supporting Information). In contrast, overexpressing endogenous PP2Ac proteins or activating PP2A with the agonist DT‐061 effectively reduced ERK signaling, even in the presence of AZD4573 (Figure S3F,G, Supporting Information). Furthermore, the phosphate release assay revealed a decline in PP2Ac phosphatase activity following an initial induction and a significant decrease after 8 h of AZD4573 treatment (Figure 3J). All these findings suggested that after blocking the inhibitory phosphorylation by CDK9, PP2Ac might undergo re‐inhibition mediated by other regulatory factors and subsequently contribute to ERK phosphorylation. It was noteworthy that in addition to attenuating threonine phosphorylation of PP2Ac (Figure S2C, Supporting Information), CDK9i augmented its overall tyrosine phosphorylation (Figure S3H, Supporting Information). As previously reported, the tyrosine kinase Src exerts inhibitory effects on PP2Ac through phosphorylation at Y127, Y284, and Y307, among which Y284 is a major contributor to Src‐induced ERK activation.^[^ ^26^ ^]^ Our data further indicated that overexpressing WT Src led to the phosphorylation of both PP2Ac and ERK, while Src knockdown or the expression of inactive mutations: K298M‐Y419F‐Y530F (3M) triple mutant Src or kinase‐dead (KD) Src failed to elicit these effects (Figure 3K,L). An in vitro phosphatase assay demonstrated that basal PP2Ac activity was significantly enhanced in cells ectopically expressing 3M‐Src or KD‐Src, but markedly decreased in cells expressing WT‐Src (Figure S3I, Supporting Information). Moreover, the PP2Ac Y284F mutant, which was incapable of being phosphorylated and repressed by Src, caused the dephosphorylation of ERK and counteracted Src‐ or CDK9i‐induced ERK activation (Figure 3M; Figure S3J, Supporting Information). In addition, Src itself serves as a crucial upstream kinase in the regulation of the ERK/MAPK pathway.^[^ ^27^ ^]^ Depletion of Src reduced p‐ERK and abrogated the induction by CDK9i (Figure S3K, Supporting Information), underscoring the indispensable role of Src activity within the dynamic CDK9/PP2Ac/ERK network. Src expression was positively correlated with KRAS mutation or expression in LUAD, COAD, or PAAD patients (Figure S3L,M, Supporting Information). By targeting both regulatory mechanisms of PP2Ac, cotreatment with CDK9i and Srci (Dasatinib) demonstrated superior anti‐proliferative activity compared to treatment with either drug alone (Figure S3N, Supporting Information). This combination synergistically attenuated ERK signaling and promoted cellular apoptosis (Figure S3O,P, Supporting Information).

The CDK9 interactome identified above revealed potential associations among CDK9, PP2Ac, Src, and ERK (Figure S3Q, Supporting Information). The reinforced signals of all three proteins in the anti‐CDK9 immunoprecipitates from AZD4573‐treated cells suggested an induced interaction between CDK9 and the Src/PP2Ac/ERK complex (Figure 3N). Additionally, reciprocal endogenous co‐IPs conducted with anti‐Src or anti‐ERK antibodies also confirmed these interactions (Figure 3O). CDK9 depletion significantly attenuated the binding of the complex (Figure 3P). Notably, a remarkable induction in Src and p‐Src was detected within the purified nuclei prepared from AZD4573‐treated SW620 and MIA cells, accompanied by increased levels of p‐ERK, PP2Ac, p‐c‐Jun, and p‐STAT3 within both the nucleus and cytoplasm (Figure 3Q). Furthermore, the immunofluorescence assay also revealed an accumulated co‐location between p‐ERK/p‐Src and PP2Ac in the nucleus following CDK9 inhibition (Figure 3R). Collectively, these data propose a model whereby CDK9/Src interplay acts as an inhibitory mechanism for PP2Ac, while CDK9 inhibition facilitates the recruitment of Src to re‐suppress PP2Ac, leading to activation of ERK and partial restoration of Pol II CTD phosphorylation. This cooperative regulation, along with other transcription elongation complexes (TEC), effectively modulates transcription elongation events (Figure 3S).

### 2.4 Blocking the ERK/MAPK Pathway Enhances the Efficacy of CDK9i

**Figure 4:**
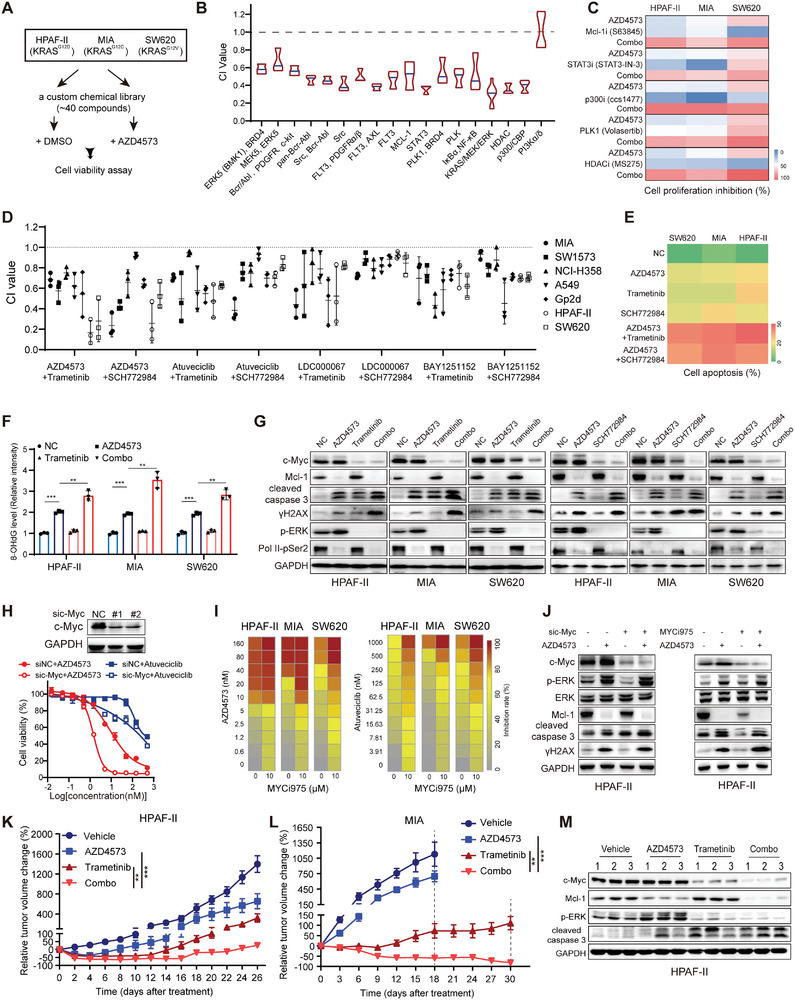
Concurrent perturbation of CDK9 and ERK/c‐Myc signaling demonstrates synergistic response in KRAS‐mutant cancers. A) Schematic depiction of the compound screening to identify molecules that enhanced the anti‐proliferative effect of AZD4573. Proliferation inhibition was monitored by an SRB assay after 72 h of treatment. Data are representative of three independent experiments. B) Bar plot showing the CI values of the compounds targeting the indicated proteins with AZD4573 using CalcuSyn. C) Heatmap showing the cell proliferation inhibition of HPAF‐II, MIA, and SW620 cells treated with AZD4573 (10 nm) and the listed compounds (10 nm for Volasertib and 1 µm for the others), alone or in combination for 72 h (shown are mean values, *n =* 3). D) CI values of different CDK9i (AZD4573/Atuveciclib/LDC000067/Bay1251152) and Trametinib/SCH772984 in various KRAS‐mutant cancer cell lines. E) Cells treated with AZD4573 (40 nm) and Trametinib/SCH772984 (100 nm) for 48 h were stained with Annexin V/PI followed by flow cytometry (shown are mean values, *n =* 3). F) The 8‐OHdG levels in the cells treated as described above were analyzed by cell immunofluorescence. G) Western blot analysis of cell lines HPAF‐II, MIA, and SW620 cells treated with 40 nm AZD4573 and 100 nm Trametinib/SCH772984 for 24 h. H) Dose‒response curves of c‐Myc knockdown HPAF‐II cells treated with CDK9i (AZD4573 or Atuveciclib). Data are presented as mean ± SD. I) Heatmap showing the proliferation inhibition of HPAF‐II, MIA, and SW620 cells treated with the indicated CDK9i and MYCi975 (10 µm) for 72 h (shown are mean values, *n =* 3). J) HPAF‐II cells treated with 40 nm AZD4573 plus c‐Myc knockdown or inhibitor (MYCi975, 10 µm) for 24 h were assessed by immunoblotting. Relative tumor volume (RTV) change (%) of HPAF‐II K) and MIA cell L) xenografts treated with vehicle, AZD4573 (10 mg kg^−1^, BID q2h, 2 days on/5 days off), or Trametinib (0.2 mg kg^−1^ per day), alone or in combination. *p < 0.05, ** *p* < 0.01, ***p < 0.001 by one‐way ANOVA with Tukey's multiple comparison test. Data are the mean changes of RTVs (%) of each group (*n =* 5); error bar: SEM. M) Western blot analyses of the tumor tissues from the above HPAF‐II xenografts. Bar plots are presented as mean ± SD (*n =* 3). P values were calculated by unpaired Student's *t*‐test F). *p < 0.05, **p < 0.01 and ***p < 0.001.

Given the compensatory responses to CDK9i in KRAS‐mutant cancers, we further performed high‐throughput screening (HTS) using a custom library comprising ≈40 compounds to screen combinational strategies. Three cell lines: HPAF‐II (G12D), MIA (G12C), and SW620 (G12V), were treated with each compound from this library plus AZD4573, and the combination index (CI) values were determined (**Figure** **4**A; Figure S4A, Supporting Information). Among the top hit compounds that augmented AZD4573 efficacy, seven distinct classes of inhibitors targeting KRAS/MAPK, STAT3, HDAC, p300/CBP, PLK1, Src, and Mcl‐1 exhibited no significant differences across diverse backgrounds (Figure 4B). Then, synergistic cytotoxicity was confirmed by pharmacodynamic evaluations using AZD4573 and Atuveciclib (Figure 4C; Figure S4B, Supporting Information). Notably, most of these efficacious targets, such as ERK, STAT3, HDAC2, p300, and Src, were within the CDK9 interactome identified by LC‐MS/MS, thereby potentially contributing to their synergism.

Compounds targeting the KRAS/MAPK pathway were among the most effective enhancers of CDK9i as revealed by the HTS data, further highlighting the significance of aberrant p‐ERK and recovered c‐Myc in conferring resistance to CDK9i. To confirm this, we depleted ERK1/2 and observed a significant enhancement of CDK9i efficacy compared to that of scrambled controls (Figure S4C, Supporting Information). Cotreatment of AZD4573 and MEKi (Trametinib) or ERKi (SCH772984) exhibited remarkable synergistic anti‐proliferative effects on HPAF‐II, MIA, and SW620 cell lines, as depicted by the colony formation assays (Figure S4D, Supporting Information). Additionally, other CDK9i including Atuveciclib, BAY 1251152, and LDC000067 were evaluated for their synergistic effects with MEKi/ERKi on various KRAS‐mutant cancer cell lines, with CI values less than 1 (Figure 4D). The synergistic effect was demonstrated by enhanced cell apoptosis and DNA damage, as evidenced by elevated levels of cleaved caspase‐3, 8‐OHdG, and γH2AX, accompanied by a potent downregulation of c‐Myc and Mcl‐1 (Figure 4E–G; Figure S4E, Supporting Information). Consistently, c‐Myc knockdown markedly sensitized cells to CDK9i treatment (Figure 4H). MYCi975, a c‐Myc inhibitor, in conjugation with CDK9i, exhibited synergistic anti‐proliferative effects (Figure 4I; Figure S4F, Supporting Information). These combinations also resulted in increased levels of cleaved caspase‐3 and γH2AX (Figure 4J).

We subsequently assessed the biological implications of combined CDK9/ERK inhibition in two KRAS‐mutant cell‐derived xenograft (CDX) models: HPAF‐II and MIA. Compared to the individual administration of AZD4573 or Trametinib, which showed only minor or moderate anti‐tumor effects respectively, the drug combination significantly inhibited tumor growth without causing noteworthy weight loss (Figure 4K,L; Figure S4G, Supporting Information), concomitant with augmented cellular apoptosis, as evidenced by elevated levels of cleaved caspase‐3 in the tumor lysates from the co‐administered group (Figure 4M). In summary, concurrent targeting of CDK9 and the feedback activation of ERK‐c‐Myc signaling exhibits synergistic cytotoxicity, which pronouncedly promotes mitochondrial dysfunction and cell apoptosis in vitro, and effectively suppresses the growth of KRAS‐mutant tumors in vivo.

### 2.5 CDK9i Synergize with KRAS Signaling Inhibitors to Arrest KRAS‐mutant Cancers and Overcome KRASi Resistance

**Figure 5:**
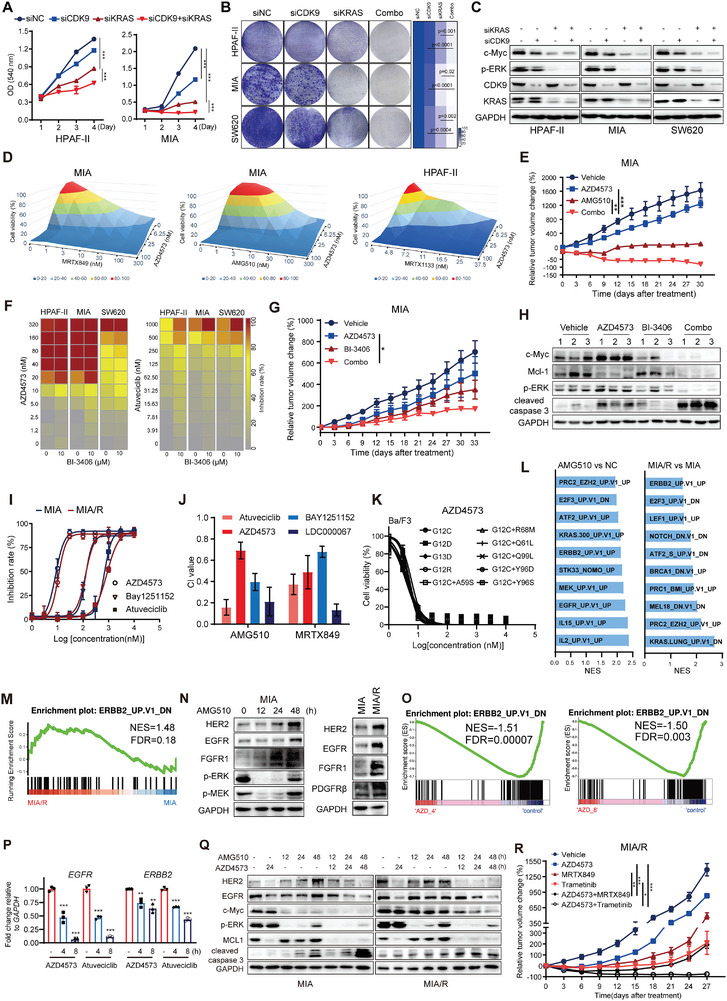
Combined inhibition of CDK9 and SOS1/KRAS signaling induces synergistic cytotoxicity in KRAS‐mutant cancers and overcomes resistance to KRASi. A) Relative cell proliferation of HPAF‐II and MIA cells treated with siCDK9 and siKRAS, alone or in combination for 4 days (mean ± SD, *n =* 3). B) Cells transfected with CDK9 or KRAS siRNAs, alone or in combination were plated and clonogenic growth was monitored at 7–10 days post‐transfection. C) Cells treated as described above were collected 48 h post‐transfection and subjected to western blotting for c‐Myc and p‐ERK. D) Heatmaps showing the effects of the combination of AZD4573 with AMG510/MRTX849 or MRTX1133 on MIA or HPAF‐II cells, respectively for 72 h (shown are mean values, *n =* 3). E) RTV change (%) of MIA xenografts treated with AZD4573 (7.5 mg kg^−1^, BID q2h, 2 days on/5 days off), or AMG510 (20 mg kg^−1^ per day), alone or in combination for 30 days. *n =* 5; error bar = SEM. F) Heatmap showing cell proliferation inhibition of HPAF‐II, MIA and SW620 cells treated with the indicated CDK9i and BI‐3406 for 72 h (shown are mean values, *n =* 3). G) RTV change (%) of MIA xenografts treated with AZD4573 (7.5 mg kg^−1^, BID q2h, 2 days on/5 days off), BI‐3406 (25 mg kg^−1^/BID), alone or in combination for 33 days; *n =* 5; error bar = SEM. H) Tumors from mice treated as described in G) were harvested after treatments, and lysates were analyzed by blotting for the indicated proteins. I) MIA parental and resistant cell lines were treated with CDK9i (AZD4573, Bay1251152, and Atuveciclib) at the indicated concentrations. Cell proliferation inhibition was assessed by SRB assays at 72 h. J) CI values of different CDK9i plus AMG510/MRTX849 in MIA/R cell lines. K) Relative cell viability of Ba/F3 cells expressing the indicated KRAS mutations treated with a concentration gradient of AZD4573 for 72 h. L) Significantly enriched gene sets identified by GSEA using oncogenic signatures in KRAS^G12C^i‐treated or KRAS^G12C^i‐resistant cells. M) GSEA plots of gene sets involved in ERBB2 signaling in MIA/R cells. N) Western blot analysis of MIA cells incubated with AMG510 for the indicated times or compared with MIA/R cells. O) GSEA plots of gene sets involved in ERBB2 signaling in HPAF‐II cells treated with AZD4573 for 4 or 8 h. P) RT‐qPCR analysis of *EGFR* and *ERBB2* mRNA levels in HPAF‐II cells exposed to AZD4573 (40 nm) or Atuveciclib (1 µm) for the indicated times. Data are presented as mean ± SD (*n =* 3). *p < 0.05, **p < 0.01, ***p < 0.001 by unpaired Student's *t* test. Q) Immunoblot assays of indicated protein levels in MIA and MIA/R cells treated with AZD4573 (40 nm) in combination with AMG510 (100 nm) for 24 or 48 h. R) RTV change (%) of MIA/R xenografts treated with AZD4573 (15 mg kg^−1^, BID q2h, 2 days on/5 days off), alone or in combination with MRTX849 (10 mg kg^−1^per day)/Trametinib (0.2 mg kg^−1^per day), for 27 days (*n =* 5); error bar = SEM.

Considering the aforementioned observation that inactivation of ERK sensitized cells to CDK9i, we reasoned that targeting the upstream KRAS network could also be an actionable strategies to abrogate CDK9i tolerance. Simultaneous knockdown of both KRAS and CDK9 led to a synergistic inhibition of cell proliferation and colony formation (**Figure** **5**A,B), accompanied by a more marked reduction in c‐Myc and p‐ERK (Figure 5C). Accordingly, AZD4573 in combination with AMG510/MRTX849 (KRAS^G12C^i) or MRTX1133 (KRAS^G12D^i) synergistically arrested cell proliferation, impaired cell clonality, and enhanced apoptosis and DNA damage (Figure 5D; Figure S5A–C, Supporting Information). The in vivo anti‐tumor efficacy of CDK9i plus KRASi was evaluated using the MIA xenograft model. AZD4573 combined with AMG510 completely eradicated tumors below palpable detection in 50% of the mice, exhibiting a superior TGI rate compared to that of each monotherapy (Figure 5E), with no significant weight loss (Figure S5D, Supporting Information).

Although our data suggested that KRAS signaling inhibitors can serve as promising combination partners for CDK9i, recent studies have proposed targeting the guanine exchange factor son of sevenless 1 (SOS1), a node that positively modulates KRAS kinase activity, as a potential therapeutic strategy for KRAS‐mutant cancers. Accordingly, the combination of SOS1i (BI‐3406) with either AZD4573 or Atuveciclib yielded robust synergistic anti‐proliferative effects on MIA, HPAF‐II, and SW620 cell lines (Figure 5F). Dual inhibition of SOS1 and CDK9 reduced cell clonality and enhanced cell apoptosis (Figure S5E–G, Supporting Information). Moreover, co‐administration of BI‐3406 and AZD4573 was well tolerated (Figure S5H) and caused substantial tumor suppression in the entire cohort of MIA tumor‐bearing mice (Figure 5G). The levels of c‐Myc and Mcl‐1 were strongly reduced in tumor tissues from the combined group, together with a noticeable increase in caspase‐3 cleavage (Figure 5H).

Additionally, we previously reported that p‐ERK could be indistinguishably inhibited by KRAS^G12C^i even in a MIA KRAS^G12C^i‐resistant (MIA/R) cell line.^[^ ^11^ ^]^ This led to the hypothesis that KRASi could potentiate the effect of CDK9i by reducing p‐ERK in KRASi‐resistant cells, as well. We first revealed that CDK9i significantly halted the proliferation of both MIA parental and resistant cell lines (Figure 5I). Additionally, CDK9i in conjunction with KRAS signaling inhibitors exhibited a strong synergism on MIA/R cells (Figure 5J) and produced a durable suppression in cell clonality (Figure S5I, Supporting Information). These cotreatments resulted in additional reductions of p‐ERK, c‐Myc, and Mcl‐1, and enhanced caspase‐3 cleavage and DNA damage (Figure S5J, Supporting Information). Moreover, AZD4573 alone potently inhibited the proliferation of Ba/F3 cells expressing KRAS^G12C^, KRAS^G12D^, or second‐site mutants that have been reported to confer acquired resistance to KRAS^G12C^i^[^ ^28^ ^]^ (Figure 5K).

The compensatory activation of wild‐type KRAS through multiple receptor tyrosine kinases (RTKs) renders site‐specific KRAS‐mutant inhibitors less effective.^[^ ^29^ ^]^ GSEA analysis showed that AMG510 post‐treatment cells presented a significant upregulation of ERBB2 and EGFR signaling enrichment, demonstrating overall concordance in the transcriptional profile of MIA/R cells (Figure 5L,M). Immunoblot analysis confirmed the elevated protein levels of EGFR, FGFR1, and HER2 in post‐treatment or resistant cells relative to pre‐treatment or parental cells (Figure 5N). A window trial revealed that two days of KRASi treatment induced an adaptive bypass response characterized by increased RTKs and reactivated MEK/ERK, which might contribute to resistance against sustained KRAS inhibition.

The dynamic formation of BRD4‐based complexes reportedly enables P‐TEFb pause‐release and transcriptional elongation following Trametinib treatment.^[^ ^30^ ^]^ We thus surmised that targeting CDK9 would impede the adaptation by RTKs. Negative enrichment of ERBB2, VEGF, and PGF signaling was observed in AZD4573‐treated HPAF‐II cells (Figure 5O; Figure S5K, Supporting Information). Inhibition of CDK9 effectively suppressed the transcriptional adaptive genes such as *EGFR* and *ERBB2* (Figure 5P). The synergistic efficacy of KRASi and CDK9i was further evaluated to reveal their impact on modulating the RTK‐KRAS‐MAPK cascade. Cotreatment with CDK9i and KRAS^G12C^i or KRAS^G12D^i successfully blocked the upregulation of RTKs and rebound of p‐ERK (Figure 5Q; Figure S5L, Supporting Information). Notably, the compensatory expression of Mcl‐1, which is known to be induced by various tyrosine kinase inhibitors (TKIs) including MEK, EGFR, MET, and HER2,^[^ ^31^ ^]^ was also substantially attenuated by CDK9i during combination therapy. Furthermore, co‐administration of AZD4573 and Trametinib or MRTX849 at well‐tolerated doses produced sustained growth inhibition in MIA/R xenografts, surpassing the partial effects of each monotherapy alone (Figure 5R; Figure S5M, Supporting Information). Overall, the simultaneous inhibition of CDK9 and KRAS signaling resulted in the synergistic abrogation of adaptive RTKs and Mcl‐1, as well as ERK‐MYC activation. This combination amplifies both the magnitude and duration of each individual agent and indicates reciprocal antagonism against drug resistance.

### 2.6 CDK9i‐induced ERK Activation Accounts for Complement Activation and Tumor Microenvironment Remodeling

**Figure 6:**
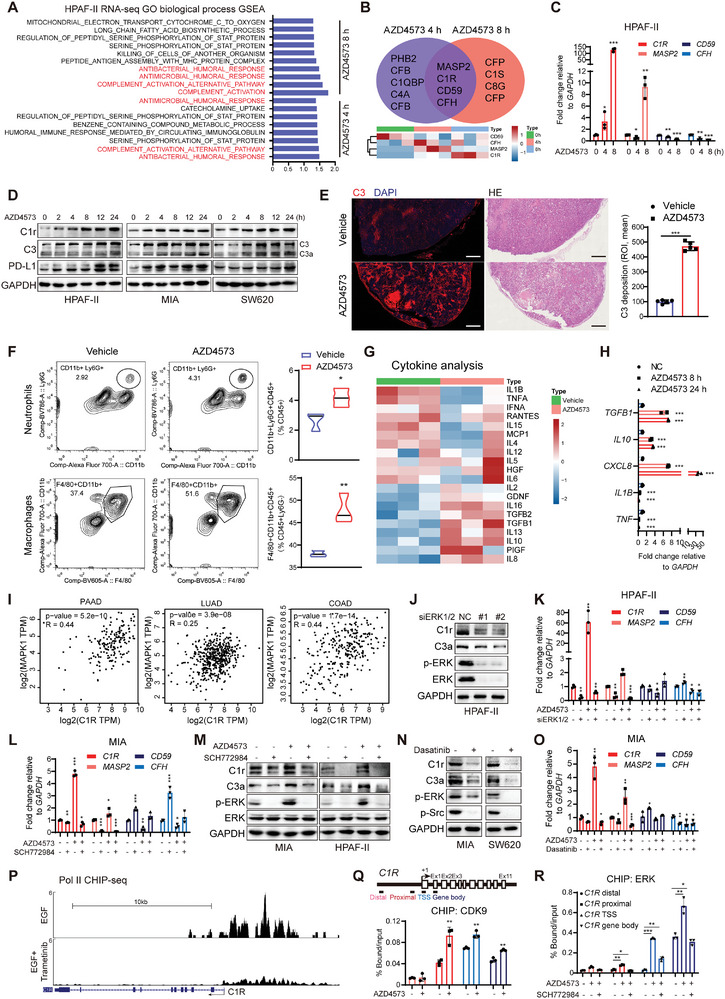
CDK9 inhibition‐induced ERK signaling is associated with increased complement activation. A) GSEA using the MsigDB Gene Ontology (GO) biological process signature identified pathways (Padj < 0.05) that were upregulated after AZD4573 treatment. B) The Venn diagram was used to identify commonly deregulated genes associated with complement activation after AZD4573 (40 nm) treatment for 4 h and 8 h (up). Heatmap showing the overlapping deregulated genes (down). C) Bar plots showing relative expression levels of complement genes after AZD4573 (40 nm) treatment for 4 or 8 h in HPAF‐II determined by RT‐qPCR. D) Immunoblot showing C1r, C3, and PD‐L1 in HPAF‐II, MIA, and SW620 cells after AZD4573 (40 nm) treatment for the indicated times. E) C3 deposition in vehicle‐ or AZD4573‐treated (15 mg kg^−1^ for 21 days) HPAF‐II tumor tissues were observed by IF staining (scale bar: 1 mm). F) Analysis of the leukocyte infiltration into the above vehicle‐ or AZD4573‐treated HPAF‐II tumor tissues by FACS. Total leukocytes (CD45^+^), macrophages (F4/80^+^/CD11b^+^), and neutrophils (CD11b^+^/Ly6G^+^) were analyzed. G) Cytokine analyses of the homogenates from the above tumor tissues (*n =* 3). H) The expression of the indicated cytokines was analyzed by RT‐qPCR in HPAF‐II cells treated with 40 nm AZD4573 for 8 or 24 h. I) Correlations between C1R and MAPK1 in the TCGA PAAD, LUAD, and COAD cohorts were determined with Pearson's correlation coefficients. J) Immunoblot showing C1r and C3a in HPAF‐II cells after ERK1/2 knockdown. K) Bar plots showing the relative expression levels of complement genes after AZD4573 (40 nm) treatment for 8 h in ERK‐knockdown HPAF‐II cells determined by RT‐qPCR. L) Bar plots showing the expression levels of complement genes determined by RT‐qPCR in MIA cells treated with AZD4573 plus SCH772984 for 12 h. M) Immunoblots for the indicated proteins in MIA and HPAF‐II cells treated with AZD4573 (40 nm) plus SCH772984 (100 nm) for 12 h. N) Immunoblots for the indicated proteins in MIA and SW620 cells treated with 100 nm Dasatinib for 12 h. O) The mRNA levels of complement genes in MIA cells treated with AZD4573 (40 nm) and Dasatinib (100 nm) for 12 h were determined by RT‐qPCR. P) Representative Pol II ChIP‐seq signal (*C1R* locus) in EGF‐stimulated A549 cells treated with or without Trametinib for 3 h (GSE85089). Q) Schematic diagrams of the *C1R* genomic locus. Boxes represent exons and bars below the genes show the positions of amplicons used for CHIP‐qPCR analyses. Distribution of CDK9 on the *C1R* gene locus was assessed after AZD4573 treatment (40 nm, 8 h) in HPAF‐II cells. R) ChIP‐qPCR assays were performed with an anti‐ERK antibody using chromatin prepared from HPAF‐II cells treated with AZD4573 (40 nm) alone or in combination with SCH772984 (100 nm) for 8 h. Data are presented as mean ± SD (*n =* 3). *p < 0.05, **p < 0.01, ***p < 0.001 by unpaired Student's *t* test.

To elucidate the insufficient responsiveness of KRAS‐mutant cancers to CDK9i in nude mouse xenograft models, we next investigated the impact of CDK9i on the tumor microenvironment (TME). KEGG analysis of the DEGs in HPAF‐II cells treated with AZD4573 revealed enrichment of genes related to cytokine‐cytokine receptor interactions and ECM‐receptor interactions (Figure S6A, Supporting Information). In addition, GSEA using HALLMARK gene sets revealed marked positive enrichment in the complement activation in cells treated with AZD4573 for both 4 and 8 h (**Figure** **6**A). We then performed Venn diagram analysis and identified four overlapping genes associated with the complement system: *C1R*, *MASP2*, *CD59*, and *CFH* (Figure 6B). The expression of complement activators *C1R* and *MASP2* was significantly upregulated at both time points compared to that in the control group; whereas complement inhibitory proteins *CD59* and *CFH* were downregulated (Figure 6C; Figure S6B, Supporting Information). C3 and its active fragment C3a, downstream effectors of classical complement activation mediated by C1q/C1s/C1r, were time‐dependently increased following AZD4573 treatment, as well as PD‐L1 in KRAS‐mutant cancer cells (Figure 6D). Furthermore, C3 deposition in tumor tissues showed a higher abundance in mice treated with AZD4573 than in vehicle control mice (Figure 6E). These findings suggest that inhibition of CDK9 triggers complement activation mediated by C1r/C3 signaling.

Complement activation in the TME orchestrates the recruitment of myeloid‐derived suppressor cells (MDSCs), neutrophils, and tumor‐associated macrophages (TAMs), establishing a pro‐survival milieu.^[^ ^32^ ^]^ Analysis via the TIMER database revealed significant positive correlations between the expression of C1r or C3 and M2‐macrophage infiltration in PAAD or LUSC patients (Figure S6C, Supporting Information). Subsequently, we detected the constitution of innate immune cells and cancer‐related inflammatory factors in vivo. The proportions of F4/80^+^/CD11b^+^ macrophages and Ly6G^high^/CD11b^+^ neutrophils among CD45^+^ cells recruited to the tumor regions were found to be higher in AZD4573‐treated mice than in vehicle‐treated controls (Figure 6F; Figure S6D, Supporting Information). Likewise, the levels of chemokines and cytokines (e.g., IL8; IL10, and TGF‐β) known to favor immune‐suppressive myeloid cells^[^ ^33^ ^]^ were significantly elevated in AZD4573‐treated tumor homogenates (Figure 6G). Additionally, CDK9i evoked the expression of these genes while decreasing those associated with inflammation and tumor killing (e.g., *TNF* and *IL1B*) (Figure 6H).

Correlation analysis using global *C1R* transcription per million (TPM) data revealed a significant positive association with *MAPK1* TPM levels in PAAD, COAD, and LUAD patients (Figure 6I). To investigate the possible involvement of p‐ERK in regulating C1r/C3, knockdown of ERK1/2 or inhibition of ERK with SCH772984 markedly reduced the protein levels of C1r and C3a, and abrogated the increase in the transcription of *C1R* and *MASP2* induced by AZD4573 (Figure 6J–M). These results contrasted with the effects observed in PP2Ac‐knockdown or Calyculin A (PP2A inhibitor)‐treated cells (Figure S6E–G, Supporting Information), but agreed well with those observed following Src knockdown or Dasatinib (Src inhibitor) treatment (Figure 6N,O; Figure S6H,I, Supporting Information). To examine the impact of the CDK9/ERK module on *C1R* transcriptional regulation, we analyzed the deposited data (GSE85089) and found that in the context of an acute EGF‐response, Trametinib treatment decreased Pol II levels across the enhancer, TSS and gene body regions of *C1R* (Figure 6P). We then conducted CHIP‐qPCR analyses and found that AZD4573 treatment significantly enhanced the occupancy of CDK9 and ERK at the TSS and gene body regions of *C1R*, while SCH772984 markedly attenuated the ERK density at the *C1R* locus (Figure 6Q,R). Collectively, these data underscore the pivotal role of CDK9i‐induced ERK activation in regulating *C1R* transcriptional regulation.

### 2.7 Co‐targeting CDK9 and the Complement System Potently Suppresses KRAS‐mutant Tumors In Vivo

**Figure 7:**
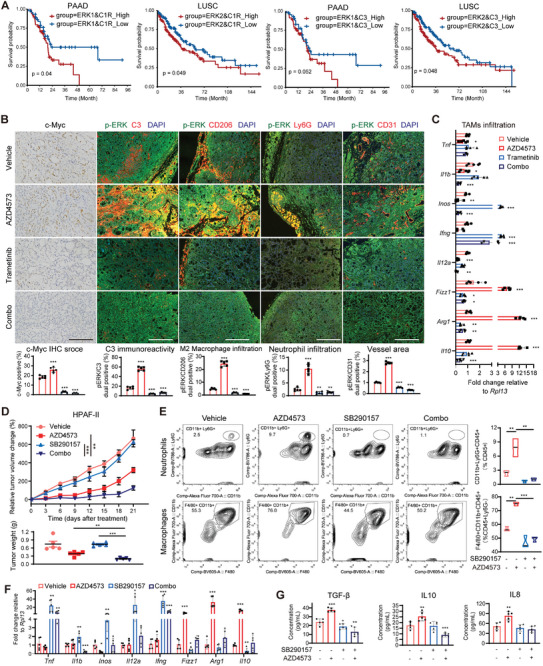
Combination therapy targeting CDK9 and the complement system exerts a robust anti‐tumor effect and impairs the immunosuppressive TME. A) Cumulative survival curves according to ERK1/ERK2 and C1R/C3 expression in PAAD and LUSC patients in the TCGA database. B) Representative IHC/IF images of c‐Myc, p‐ERK, C3, CD206, Ly6G, and CD31 expression in the tumor tissues of HPAF‐II xenografts treated with AZD4573 plus Trametinib (from Figure 4K). The quantification of dual‐positive cells (%) among total positive cell types is shown (scale bars, 100 µm). C) RT‐qPCR analysis of selected M1 and M2 markers of macrophages infiltrating the above HPAF‐II xenografts treated with AZD4573 plus Trametinib. *p < 0.05, **p < 0.01, ***p < 0.001, unpaired Student's *t* test. Data were relative to *Rpl13* expression and normalized versus the mean of the vehicle group (mean ± SD, *n =* 5). D) RTV changes (%) and tumor weights of HPAF‐II xenografts treated with vehicle, AZD4573 (15 mg kg^−1^, BID q2h, 2 days on/5 days off), SB290157 (5 mg kg^−1^, 6 days on/1 day off), or the combination of both drugs (*n =* 5); error bar = SEM. E) Analysis of the leukocyte infiltration in HPAF‐II tumor tissues treated as described above by FACS. Total leukocytes (CD45^+^), macrophages (F4/80^+^/CD11b^+^), and neutrophils (CD11b^+^/Ly6G^+^) were analyzed (*n =* 3). F) RT‐qPCR analysis of selected M1 and M2 markers of macrophages infiltrating HPAF‐II xenografts treated with AZD4573 plus SB290157 (*n =* 5). G) Local TGF‐β, IL10, and IL8 in the above HPAF‐II tumors were assessed by ELISA kits (*n =* 5). Bar plots are presented as mean ± SD. *p < 0.05, **p < 0.01, ***p < 0.001 by unpaired Student's *t*‐test.

To determine the possible contributions of C1r and C3 to cancer, we investigated the alteration frequency of *C1R* gene using the cBioPortal database. Significant amplification or mutation events are observed in pancreatic, colorectal, and lung cancers, with minimal alterations in hematologic malignancies such as AML (Figure S7A, Supporting Information), indicating a potential role for C1r in solid tumor progression. High expression of C1R/C3 is associated with high pathological grade (Figure S7B, Supporting Information) and poor prognosis in PAAD, COAD, and lung cancer patients (Figure S7C, Supporting Information). Furthermore, we developed a better prognostic indicator by using the expression pattern of C1R/C3^high^ERK1^high^ for PAAD patients, and C1R/C3^high^ERK2^high^ for LUSC patients (**Figure** **7**A). We then characterized the cells presenting p‐ERK and complement activation through double immunofluorescence staining of tumor tissues (Figure 7B). Cytoplasmic p‐ERK signal was observed in both tumor cells and infiltrating cells. Confocal microscopy revealed robustly enhanced C3 immunoreactivity in areas overlapping with high p‐ERK sites in AZD4573‐treated tumors compared to vehicle‐treated tumors where C3 deposition was negligible, demonstrating a significant positive correlation between C3 and p‐ERK. Cotreatment with Trametinib diminished p‐ERK and C3 deposition induced by AZD4573. This observed effect was consistent with changes in the c‐Myc immunohistochemistry (IHC) score. Notably, AZD4573 treatment facilitated tumor angiogenesis, as indicated by increased CD31‐labeled vascular endothelial cells, which exhibited a positive correlation with p‐ERK staining.

The complement system has been demonstrated to promote macrophage polarization toward an immunosuppressive M2‐like phenotype.^[^ ^34^ ^]^ We observed a higher density of CD206^+^ macrophages and Ly6G^+^ neutrophils in the p‐ERK‐high tumor regions (Figure 7B), especially in the xenografts after treatment with AZD4573. To further characterize the phenotype of TAMs, CD11b^+^/Ly6C^−^ macrophages were sorted to >98% purity to analyze the expression of M1‐ and M2‐like markers by gene transcription analysis. As depicted in Figure 7C, TAMs infiltrating the AZD4573‐treated tumors exhibited higher levels of M2‐like markers (particularly *Arg1*, *Fizz1*, and *Il10*) and lower levels of M1‐like markers (specifically *Il12a*, *Inos*, and *Ifng*) than did those infiltrating vehicle‐treated tumors. However, Trametinib therapy alone or in combination potently suppressed the accumulation of M2‐polarized TAMs. Collectively, these findings indicate a mutually reinforcing signaling between ERK and complement activation, highlighting their pro‐survival role by promoting angiogenesis and immunosuppression.

To investigate the translational implications, we finally evaluated the impact of pharmacological inhibition of the complement system on the efficacy of CDK9i monotherapy. We treated HPAF‐II xenografts with SB290157 trifluoroacetate salt, a C3a receptor antagonist (C3aRa), either alone or in combination with AZD4573. Remarkably, the cotreatment led to a significant reduction in tumor growth compared to each monotherapy, without any apparent signs of toxicity (Figure 7D; Figure S7D, Supporting Information). Tumor single‐cell suspensions were then subjected to FACS analysis to characterize different myeloid subsets. The infiltration of macrophages and neutrophils was elevated upon AZD4573 treatment but was significantly diminished by co‐administration with C3aRa (Figure 7E). Accordingly, RT‐qPCR analysis of the tumor homogenates showed that the AZD4573‐recruited M2 macrophages were largely eliminated by C3aRa treatment (Figure 7F). Moreover, blockade of systemic C3aR strongly suppressed the increase in the secretion of TGF‐β, IL10, and IL8, and the elevated level of C3a induced by CDK9i (Figure 7G; Figure S7E, Supporting Information), indicating there existed a tumor‐host interaction. Taken together, these data demonstrate that hindering ERK phosphorylation or complement activation holds promise for alleviating immune evasion and circumventing tumor tolerability in response to CDK9i.

## 3 Discussion

The development of tumors relies on oncogenic signals for the initiation and maintenance of tumor growth, as well as evasion of immune system‐mediated elimination. Emerging evidence indicates that perturbation of oncogenic signaling pathways may also compromise anti‐tumor immune responses, thereby contributing to resistance against molecularly targeted therapies.^[^ ^35^ ^]^ Here we demonstrated that refractory to CDK9i might be associated with compensatory ERK‐MYC activation, recovery of pro‐oncogene transcription, infiltration of pro‐tumoral TAMs and neutrophils, and tumor angiogenesis. The CDK9‐Src‐PP2A network drove rapid and persistent phosphorylation of ERK and unleashed the production of complement C1r and C3, leading to complement activation and immunosuppression. Importantly, targeting KRAS/SOS1/MEK signaling effectively counteracted this paradoxical regulation of ERK activation and improved the efficacy of CDK9i, presenting a potential means to remit the overlapping toxicity of these two drug classes. Moreover, simultaneous blockade of the complement system and CDK9 significantly impeded immune evasion and retarded tumor growth in KRAS‐mutant xenografts. These data proposed both the MAPK pathway and complement cascade as potent susceptibilities in CDK9i‐tolerant KRAS‐driven cancers.

Many cancers initially respond to targeted therapies in vitro, but genetic resistance often emerges later. However, little is understood about how tumor cells tolerate therapy in vivo before genetic resistance becomes dominant. Consistent with previous studies,^[^ ^36^ ^]^ CDK9 inhibitors showed low nanomolar cytotoxicity against KRAS mutant cells, yet their efficacy was unsatisfactory in xenograft mouse models. It's noteworthy that the depletion of CDK9 resulted in a reduction of c‐Myc and minimal inhibition of ERK, which differed from the observed effects of CDK9i. This observation suggests that CDK9, even with impaired activity, may still exert a significant influence on its interactome and gene transcription regulation. We surmise that without the recruitment by CDK9, the network of Src/PP2A/ERK collapsed, leading to an irreversible c‐Myc downregulation. Given the intricate crosstalk between CDK9 and other signaling pathways, combination therapy may offer a potential strategy for mitigating drug toxicity and overcoming tumor resistance. Preclinical studies have indicated that synergistic effects can be achieved by combining CDK9i with agents targeting BCL‐2, MDM2, PARP, BTK, EGFR, BET or BRD4, glucocorticoid receptor, and chemotherapeutic drugs such as temozolomide and 5‐fluorouracil.^[^ ^37^ ^]^ Our drug synergy studies demonstrated that co‐targeting of CDK9 and KRAS/MAPK signaling pathway eliminated ERK‐MYC activation and prevented feedback activation mediated by receptor tyrosine kinases, leading to enhanced efficacy of KRAS‐mutant cancers and overcoming KRASi resistance. We further revealed that inhibitors targeting the Src, Mcl‐1, STAT3, BRD4, p300, PLK1, or class I HDACs significantly enhanced CDK9i‐induced cell death in various KRAS‐mutant cancers. Interestingly, most of these targets coincidentally overlapped with the CDK9 interactome, potentially explaining the mechanism driving their synergy. Thus, the newly identified CDK9 interactome offers valuable insights into comprehending the intricate regulatory network of CDK9 and facilitates the exploration of innovative combinatorial strategies.

This study also identified an auxiliary phosphokinase‐phosphatase module within the Pol II‐associated regulatory complex, comprising Src, PP2Ac, and ERK, which is recruited to chromatin in response to CDK9 inhibition and resumes transcription elongation. The inhibited CDK9 reduced the phosphorylation of PP2Ac at a previously unidentified inhibitory site (T281) and recruited Src to sequentially suppress the phosphatase activity of PP2Ac through Y284 phosphorylation, resulting in sustained activation of ERK and subsequent rescue of Pol II CTD phosphorylation at Ser5. This coincided with the findings that PP2A directly dephosphorylates Pol II CTD and its depletion overrides CDK9i‐induced block,^[^ ^18^ ^]^ while ERK phosphorylates Pol II CTD at Ser5 and offers alternative pathways for transcription regulation.^[^ ^23^ ^]^ Our study detailed a spontaneous cellular feedback regulation involving Src‐mediated inactivation of PP2A, rather than artificial depletion, and activation of ERK kinases in response to CDK9i‐induced pausing. The antagonism of CDK9 kinase activity by the Src/PP2Ac/ERK complex for actively transcribing genes established a therapeutically targetable pathway in KRAS‐mutant settings. Src has been identified as a primary upstream regulator of KRAS in tumors exhibiting high p‐Src levels, thus establishing its potential as a biomarker for predicting the response to MEK/Src inhibition.^[^ ^38^ ^]^ Our study extended the finding and showed that the dual inhibition of Src and CDK9 abolished both inhibitory mechanisms of PP2Ac, thereby decreasing p‐ERK and enhancing cytotoxicity. In addition, the therapeutic potential of PP2A activators for KRAS‐driven tumors has been demonstrated, particularly when combined with MEK inhibitors.^[^ ^39^ ^]^ Concomitant PP2A agonism and CDK9 inhibition also led to augmented anti‐tumor efficacy in preclinical mouse models.^[^ ^18^ ^]^ Notably, CDK9 has been shown to catalyze the inhibitory phosphorylation of PP1 and PP4 complexes as well, forming circuits that operate at both the initiation and termination of Pol II transcription cycle.^[^ ^19^ ^]^ And BRD4, a super‐enhancers organizer, has been shown to mediate P‐TEFb release from 7SK snRNP to *MYC* and other primary response genes to compensate for sustained CDK9 inhibition.^[^ ^40^ ^]^ They propose that the induction of MYC expression by CDK9i is associated with enhanced occupancy of P‐TEFb, BRD4, Pol II, acetyl‐H3/H4 at the MYC locus, aligning with our finding that the recruitment of chromatin remodeling proteins such as BRD4 and p300 within the CDK9 interactome increased after CDK9i treatment. Moreover, as CDK9 is involved in different transcription elongation complexes,^[^ ^41^ ^]^ the underlying mechanism is multifaceted and deserves more investigations. Accordingly, our studies did not exclude the probable involvement of other kinases‐phosphatase pairs or chromatin modifiers, which may be organized in analogous switch‐like circuits, in the regulation of Pol II dynamics and efficient transcription elongation.

The efficacy of targeted agents relies not only on their ability to kill or permanently impede cancer cell proliferation but also on their potential to influence immune responses. KRAS‐mutant cancers, especially G12D‐dominated PDAC, exhibit an immunologically cold TME and heightened resistance to anti‐PD‐1 immunotherapy, presenting an intriguing avenue for investigating innovative combinatorial strategies with immunomodulators.^[^ ^42^ ^]^ Inhibition of CDK9 has recently been recognized as a promising strategy for epigenetic immunosensitization, suggesting its potential impact on awakening the “hot” tumor immunity. CDK9i monotreatment enhances the proportions of CD45^+^ immune cells and CD3^+^ T cells, activates dendritic cells, and synergistically augments immune responses when combined with checkpoint blockade.^[^ ^16^ ^]^ Our study further elucidated that CDK9i activated the complement system, specifically the C1r/C3a/C3aR axis, which emerges as an important component of tumor‐promoting inflammation, characterized by increased accumulation of educated TAMs and neutrophils. Complement activation orchestrated by C1q in TAMs and C1r/C1s/C3 in tumor cells induces PD‐L1 expression in clear‐cell renal cell carcinoma.^[^ ^43^ ^]^ Cancer cell‐derived C3 can activate C3aR on immune cells and the epithelium, ultimately modulating neutrophil infiltration and facilitating tumor metastasis to the lung or brain.^[^ ^44^ ^]^ C3aR antagonists have been reported to enhance the anticancer effectiveness of anti‐PD‐1 agents in a B16 melanoma model.^[^ ^45^ ^]^ The mechanism we have uncovered brings forward the immunoregulatory effect of targeting C3 or C3aR and confers it as a promising adjuvant treatment for targeted therapy. Interestingly, the expression of C1r and C3a was reduced in a time‐dependent manner by AZD4573 in the AML cell line MV‐4‐11, as was the downregulation of c‐Myc and p‐ERK (Figure S7F, Supporting Information), which contradicted the effects observed in solid cancers, such as PDAC. Further investigation is warranted to understand whether and how this distinct impact on the complement system and pro‐survival signaling contributes to the differential responses of hematologic tumors and solid tumors to CDK9 inhibitors in vivo. In fact, the complement system is defective in chronic lymphatic leukemia patients.^[^ ^46^ ^]^ From a translational perspective, it is imperative to dissect the versatile role of complement‐mediated tumor progression and immune modulation in KRAS‐driven and MYC‐driven cancers. In addition, our study did not exclude the potential contributions of other factors to the variable response of CDK9i in both in vitro and in vivo settings, which highlights the need for further research in immunocompetent syngeneic mouse models. Such intricacies emphasize the necessity for an ever more profound comprehension of the dynamic interactions occurring in both the TME and cellular system, thereby facilitating the rational design of highly efficient regimens that integrate targeted anticancer agents with immunotherapies.

## 4 Conclusions

In summary, our data demonstrate that simultaneous inhibition of CDK9 and the KRAS/MAPK pathway reciprocally strengthen the anticancer effect of each monotherapy in preclinical models of KRAS‐mutant cancers, including those with KRASi resistance. Moreover, the novel combination of CDK9i and complement blockade effectively abrogates immunosuppressive responses, resulting in remarkable tumor remission. Targeting the CDK9/ERK/MYC network in cancer cells, as well as their crosstalk with the TME via the complement system may yield improved responses in KRAS‐mutant patients. Further investigations into inter‐ and intra‐tumor heterogeneity are needed to identify suitable populations and improve combinatorial strategies, thus enhancing clinical outcomes.

## Conflict of Interest

The authors declare no conflict of interest.

## Author Contributions

Y. W. and L. X. contributed equally to the work. Y. W. was responsible for resources, methodology, original draft writing, editing, data curation, and conceptualization. L. X. provided resources, methodology, and conceptualization. L. L. contributed to resources and methodology. M. Y. handled writing, review, and editing. S. S. was involved in methodology and data curation. C. Y. contributed to the methodology and investigation. Y. L. provided resources and methodology. J. S. contributed resources. H. J. was responsible for project administration and conceptualization. C. X. managed writing, review, editing, project administration, data curation, and conceptualization.

## Supporting information

Supporting Information

## Data Availability

The data that support the findings of this study are available from the corresponding author upon reasonable request.
