# Supplementary material for: Unraveling the CDK9/PP2A/ERK Network in Transcriptional Pause Release and Complement Activation in KRAS‐mutant Cancers
Source: Adv Sci (Weinh). 2024 Sep 10;11(41):2404926. doi: 10.1002/advs.202404926 (PMC11538672; doi:10.1002/advs.202404926)
Supplement: Supplementary file 1 — Supporting Information [file ADVS-11-2404926-s001.pdf]

ADVANCED SCIENCE
Open Access

Supporting Information

for *Adv. Sci.*, DOI 10.1002/advs.202404926

Unraveling the CDK9/PP2A/ERK Network in Transcriptional Pause Release and Complement Activation in KRAS-mutant Cancers

*Yafang Wang, Lansong Xu, Lijun Ling, Mingyue Yao, Shangxuan Shi, Chengcheng Yu, Yingnian Li, Jie Shen, Hualiang Jiang and Chengying Xie**

Supporting Information

Unraveling the CDK9/PP2A/ERK network in transcriptional pause release and complement activation in KRAS-mutant cancers

Yafang Wang[†], Lansong Xu[†], Lijun Ling, Mingyue Yao, Shangxuan Shi, Chengcheng Yu, Yingnian Li, Jie Shen, Hualiang Jiang, Chengying Xie*

[†] These authors contributed equally.

* Correspondence to: Chengying Xie, Lingang Laboratory, 319 Yueyang Road, Shanghai 200031, China. E-mail: xiecy@lglab.ac.cn. Tel/Fax:86-021-64032758

This PDF file includes:

Figures S1 to S7

Tables S1 to S5

Supplementary materials and methods

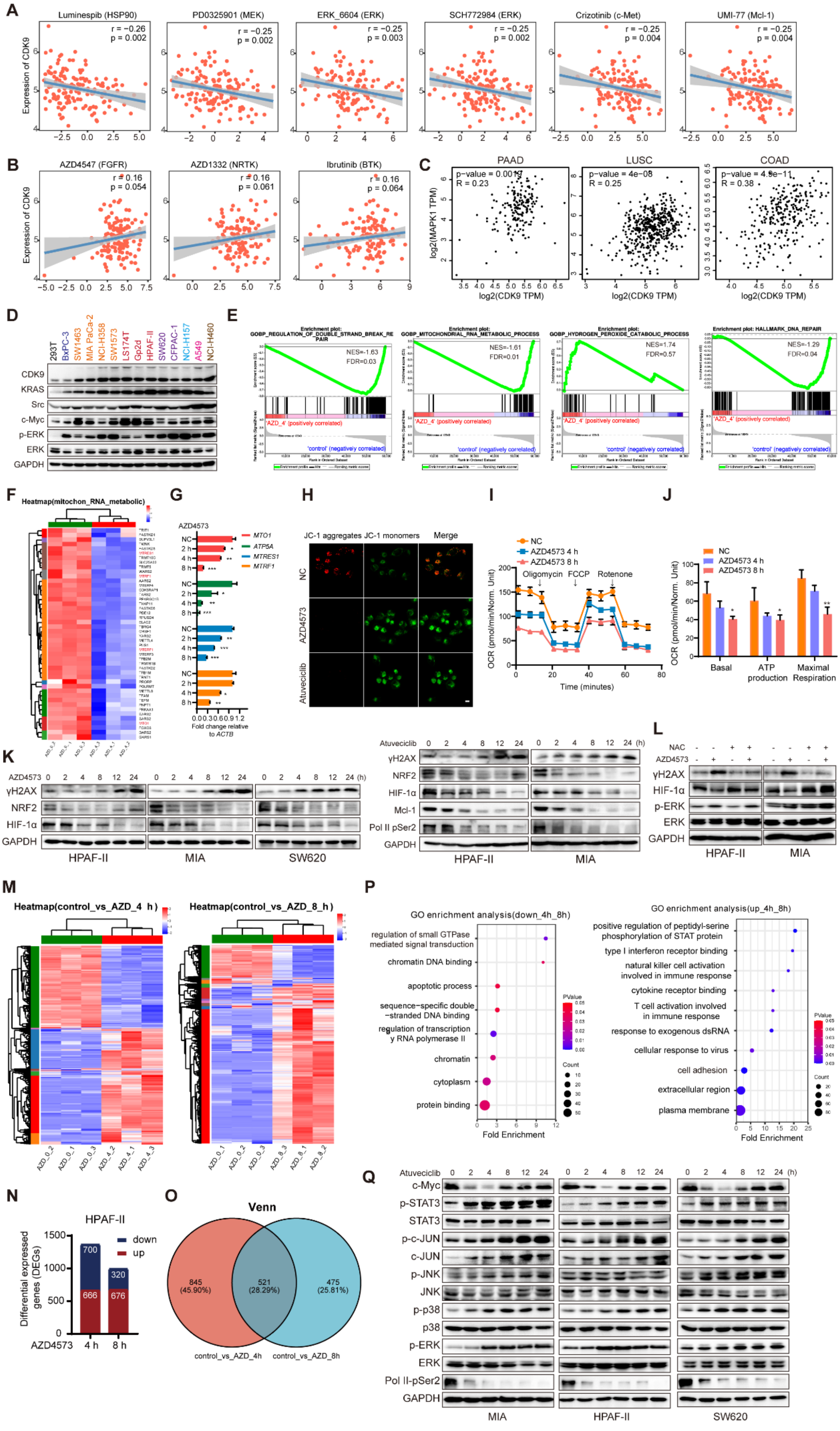
A
Luminespib (HSP90)
PD0325901 (MEK)
ERK_6604 (ERK)
SCH772984 (ERK)
Crizotinib (c-Met)
UMI-77 (Mcl-1)
Expression of CDK9
B
AZD4547 (FGFR)
AZD1332 (NRTK)
Ibrutinib (BTK)
C
PAAD
LUSC
COAD
log2(MAPK1 TPM)
log2(CDK9 TPM)
D
293T
BxPC-3
SW1463
MIA PaCa-2
NCI-H358
SW1573
LS174T
Gp2d
HPAF-II
SW620
CFPAC-1
NCI-H157
A549
NCI-H460
CDK9
KRAS
Src
c-Myc
p-ERK
ERK
GAPDH
E
F
Heatmap(mitochon_RNA_metabolic)
G
AZD4573
MTO1
ATP5A
MTRES1
MTRF1
Fold change relative to ACTB
H
JC-1 aggregates JC-1 monomers
Merge
NC
AZD4573
Atuveciclib
I
NC
AZD4573 4 h
AZD4573 8 h
Oligomycin
FCCP
Rotenone
OCR (pmol/min/Norm. Unit)
Time (minutes)
J
NC
AZD4573 4 h
AZD4573 8 h
OCR (pmol/min/Norm. Unit)
Basal
ATP production
Maximal Respiration
K
AZD4573
γH2AX
NRF2
HIF-1α
GAPDH
HPAF-II
MIA
SW620
Atuveciclib
γH2AX
NRF2
HIF-1α
Mcl-1
Pol II pSer2
GAPDH
HPAF-II
MIA
L
NAC
AZD4573
γH2AX
HIF-1α
p-ERK
ERK
GAPDH
HPAF-II
MIA
M
Heatmap(control_vs_AZD_4 h)
Heatmap(control_vs_AZD_8_h)
P
GO enrichment analysis(down_4h_8h)
regulation of small GTPase mediated signal transduction
chromatin DNA binding
apoptotic process
sequence-specific double-stranded DNA binding
regulation of transcription by RNA polymerase II
chromatin
cytoplasm
protein binding
Fold Enrichment
GO enrichment analysis(up_4h_8h)
positive regulation of peptidyl-serine phosphorylation of STAT protein
type I interferon receptor binding
natural killer cell activation involved in immune response
cytokine receptor binding
T cell activation involved in immune response
response to exogenous dsRNA
cellular response to virus
cell adhesion
extracellular region
plasma membrane
Fold Enrichment
N
HPAF-II
Differential expressed genes (DEGs)
down
up
700
666
320
676
AZD4573
4 h
8 h
O
Venn
845 (45.90%)
521 (28.29%)
475 (25.81%)
control_vs_AZD_4h
control_vs_AZD_8h
Q
Atuveciclib
c-Myc
p-STAT3
STAT3
p-c-JUN
c-JUN
p-JNK
JNK
p-p38
p38
p-ERK
ERK
Pol II-pSer2
GAPDH
MIA
HPAF-II
SW620

Figure S1 CDK9i induce mitochondrial dysfunction and transcriptome alterations in KRAS-mutant tumor cells.

(A), (B) Scatter plots showing correlation between CDK9 expression levels and AUC values of the indicated drugs in COAD, READ, PAAD, LUAD and LUSC cancer cell lines. Pearson correlation coefficients and p values are shown. (C) Pearson's correlation analysis of CDK9 and MAPK1 expressions in PAAD, LUSC and COAD patient samples from GEPIA database. (D) Expression profile of CDK9, KRAS and Src proteins in normal epithelial cell (293T) and 13 cancer cell lines harboring wildtype KRAS (BxPC-3) or mutated KRAS. (E) GSEA plots of representative gene sets involved in DNA repair, hydrogen peroxide catabolic process and mitochondrial RNA metabolic processes after CDK9 inhibition in HPAF-II cells. The normalized enrichment score (NES) and false discovery rate (FDR) are indicated. (F) Heatmap showing the downregulated genes associated with mitochondrial RNA metabolic processes in HPAF-II cells treated with 40 nM AZD4573 for 4 h compared with control. (G) RT-qPCR analysis of the indicated genes in HPAF-II cells treated with AZD4573 (40 nM) for 2, 4 or 8 h. (H) Immunofluorescence staining of JC-1 in HPAF-II cells treated with 40 nM AZD4573 or 1 μM Atuveciclib for 24 h. Red fluorescence indicates JC-1 aggregates and green fluorescence indicates JC-1 monomers. Scale bar: 10 μm. (I) OCR was measured by a Seahorse analyzer with HPAF-II cells treated with AZD4573 (40 nM) for 4 or 8 h. (J) Basal respiration, ATP production and maximal respiration were assessed, respectively. (K) Immunoblotting for γH2AX, NRF2 and HIF-1α levels in HPAF-II, MIA PaCa-2 (MIA) and SW620 cells treated with 40 nM AZD4573 or 1 μM Atuveciclib for the indicated times. (L) Western blot analysis of γH2AX, HIF-1α and p-ERK protein levels in HPAF-II and MIA cells treated with AZD4573 (40 nM, 12 h) with or without pretreatment of 5 mM NAC for 1 h. (M) All the DEGs in HPAF-II cells treated with AZD4573 for 4 or 8 h compared with control treatment (false discovery rate q-value < 0.05; fold change > 2). (N) Bar plot showing all the numbers of DEGs in HPAF-II cells treated with AZD4573 for 4 h (700 genes down versus 666 genes up) or 8 h (676 up versus 320 down). (O) Venn analysis showing the common DEGs in HPAF-II cells treated with AZD4573 for 4 or 8 h compared with control treatment. (P) GO enrichment analyses of commonly downregulated or upregulated genes are shown. (Q) Effect of Atuveciclib (1 μM) on time course expression of proto-oncogenes and IEGs. For all statistical plots, the data are presented as mean ± SD. n = 3 biological replicates. *$p < 0.05$, **$p < 0.01$, ***$p < 0.001$.

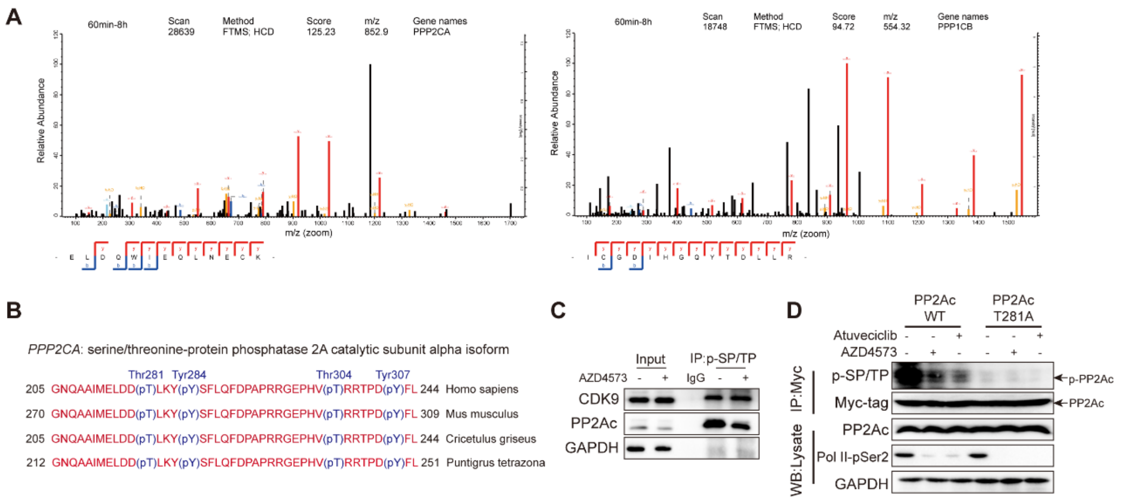

Figure S2 PP2Ac identified from the CDK9 interactome participates in the transcriptional elongation after CDK9 inhibition.

(A) The peptides of PPP2CA and PPP1B were identified in the CDK9-immunoprecipitates from AZD4573-treated HPAF-II cells using mass spectrometry. (B) PP2Ac protein sequences, including the phosphorylation sites T281, Y284, T304 and Y307, were aligned across species using BLAST. Blue letters indicate conserved serine/threonine or tyrosine residues. (C) HPAF-II cells were treated with 40 nM AZD4573 for 4 h. Phosphorylation of PP2Ac were probed with immunoprecipitants using an anti-p-SP/TP antibody. (D) MIA cells were transfected with Myc-PP2Ac WT or T281A mutant and treated with 40 nM AZD4573 or 1 μM Atuveciclib for 8 h. Phosphorylation of immunoprecipitated Myc-PP2Ac was probed with anti-p-SP/TP antibody.

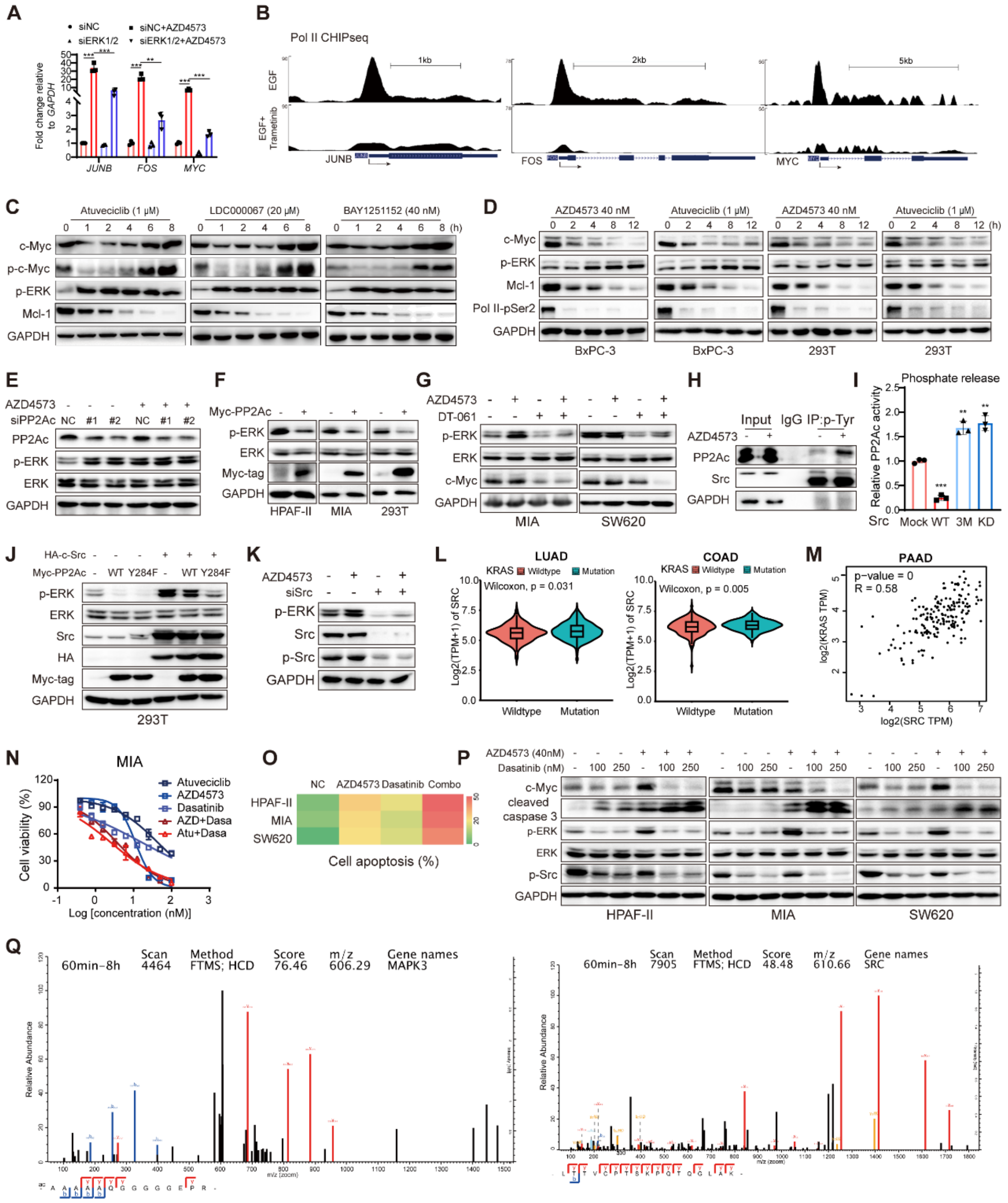

Figure S3 Src suppresses PP2Ac by phosphorylation at Y284 and promotes ERK activation.

(A) Bar plots showing expression levels of IEGs in ERK1/2-knockdown MIA cells treated with or without AZD4573 for another 8 h. (B) Representative Pol II ChIP-seq signal (*MYC*, *JUNB* and *FOS* loci) in EGF-stimulated A549 cells treated with or with Trametinib for 3 h. (C) The effect of Atuveciclib (1 μM), LDC000067 (20 μM) and Bay1251152 (40 nM) on time course expressions of c-Myc, p-c-Myc and p-ERK in MIA cells. (D) BxPC-3 and 293T cells were treated with AZD4573 (40 nM) or Atuveciclib (1 μM) for indicated times and subjected to western blotting. (E) Western

blot analyses of PP2Ac-knockdown MIA cells treated with or without AZD4573. (F) Western blot analyses of p-ERK in PP2Ac-overexpressed HPAF-II, MIA and 293T cells. (G) Cells were left untreated or pre-incubated with the PP2Ac agonist DT-061 (10 μM) for 2 h. Subsequently, cells were treated with AZD4573 for another 8 h. P-ERK and c-Myc levels were determined by western blot analysis. (H) HPAF-II cells were treated with AZD4573 for 8 h and tyrosine-phosphorylation (pTyr) status of PP2Ac was analyzed by western blot using IP with a pTyr specific antibody. (I) After transfection of Src-WT, Src-3M or Src-KD in 293T cells for 24 h, endogenous PP2Ac was immunoprecipitated and subjected to an in vitro phosphatase assay using a threonine-phosphopeptide as a substrate. (J) 293T cells were transfected with PP2Ac-WT or PP2Ac-Y284F plasmid alone or co-transfected with HA-Src-WT and subjected to western blot analysis. (K) MIA cells transfected with siSrc followed by AZD4573 treatment for another 8 h were subjected to western blot analysis. (L) Quantitative SRC expression levels in samples from KRAS WT and KRAS mutant LUAD or COAD patients. (M) Pearson's correlation analysis of SRC and KRAS expression levels in PAAD patients. (N) Cell viability of MIA cells treated with CDK9i (AZD4573 or Atuveciclib) and Dasatinib, alone or in combination. (O) Cell apoptosis in cells after co-treatment of AZD4573 (40 nM) and Dasatinib (100 nM) for 48 h was analyzed by flow cytometry after Annexin V/PI double staining (shown are mean values, n=3). (P) Immunoblot showing p-ERK and cleaved caspase-3 in cells after co-treatment of indicated AZD4573 and Dasatinib for 24 h. (Q) The peptides of MAPK3 and SRC were identified in the CDK9-immunoprecipitates from AZD4573-treated HPAF-II cells using mass spectrometry. For all statistical plots, the data are presented as mean ± SD (n = 3). ns. indicates no significance; $^{*}p < 0.05$, $^{**}p < 0.01$, $^{***}p < 0.001$ by two-tailed unpaired Student's *t* test.

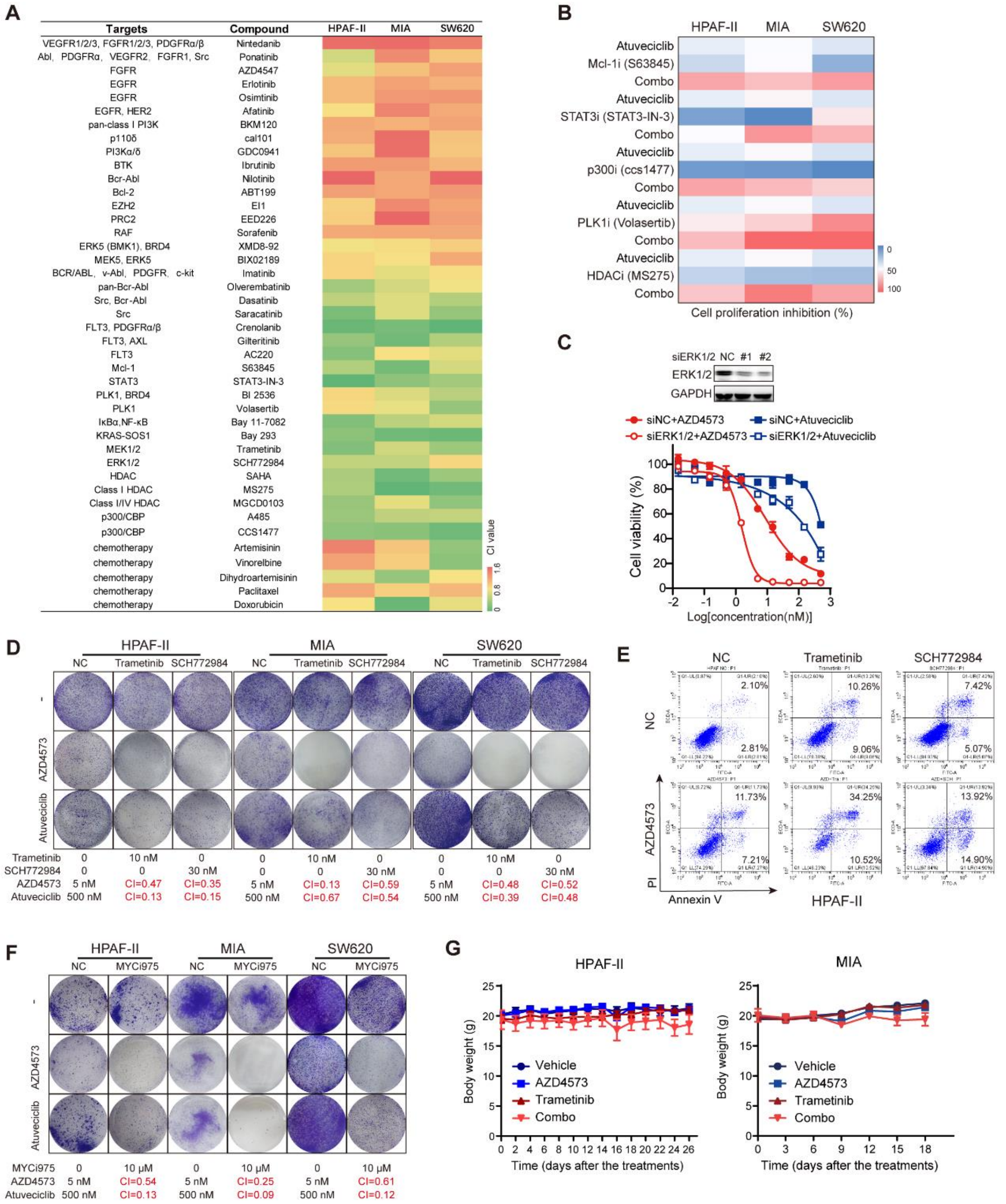

Figure S4 Combinatorial inhibition of CDK9 and ERK/c-Myc signaling synergistically arrests the growth of KRAS-mutant cancers

(A) An overview of combination index (CI) values of HPAF-II, MIA and SW620 by various pathway inhibitors plus AZD4573. CI <0.8 indicates strong synergistic effect. (B) Heatmap showing the cell proliferation inhibition of HPAF-II, MIA and SW620 cells treated with Atuveciclib (500 nM) and the listed compounds (10 nM for Volasertib and 1 μM for the others), alone or in combination for 72 h (shown are mean values, n=3). (C) Dose-response curves of cell treated with CDK9i (AZD4573 or Atuveciclib) in ERK1/2 knockdown HPAF-II cells (mean ± SD, n = 3). (D)

Clonogenic assay of HPAF-II, MIA and SW620 treated with control (DMSO), AZD4573 (5 nM)/Atuveciclib (500 nM), and Trametinib (10 nM)/SCH772984 (30 nM), alone or in combination. (E) HPAF-II cells treated with AZD4573 and Trametinib or SCH772984 for 48 h were stained with Annexin V/PI followed by flow cytometry. (F) Clonogenic assay of HPAF-II, MIA and SW620 treated with control (DMSO), AZD4573 (5 nM), and MYCi975 (10 μM), alone or in combination. (G) Mouse body weights of HPAF-II and MIA xenograft models after the co-treatment with AZD4573 and Trametinib. Data are the mean values of mouse body weight of each group (n = 5); error bar: SEM.

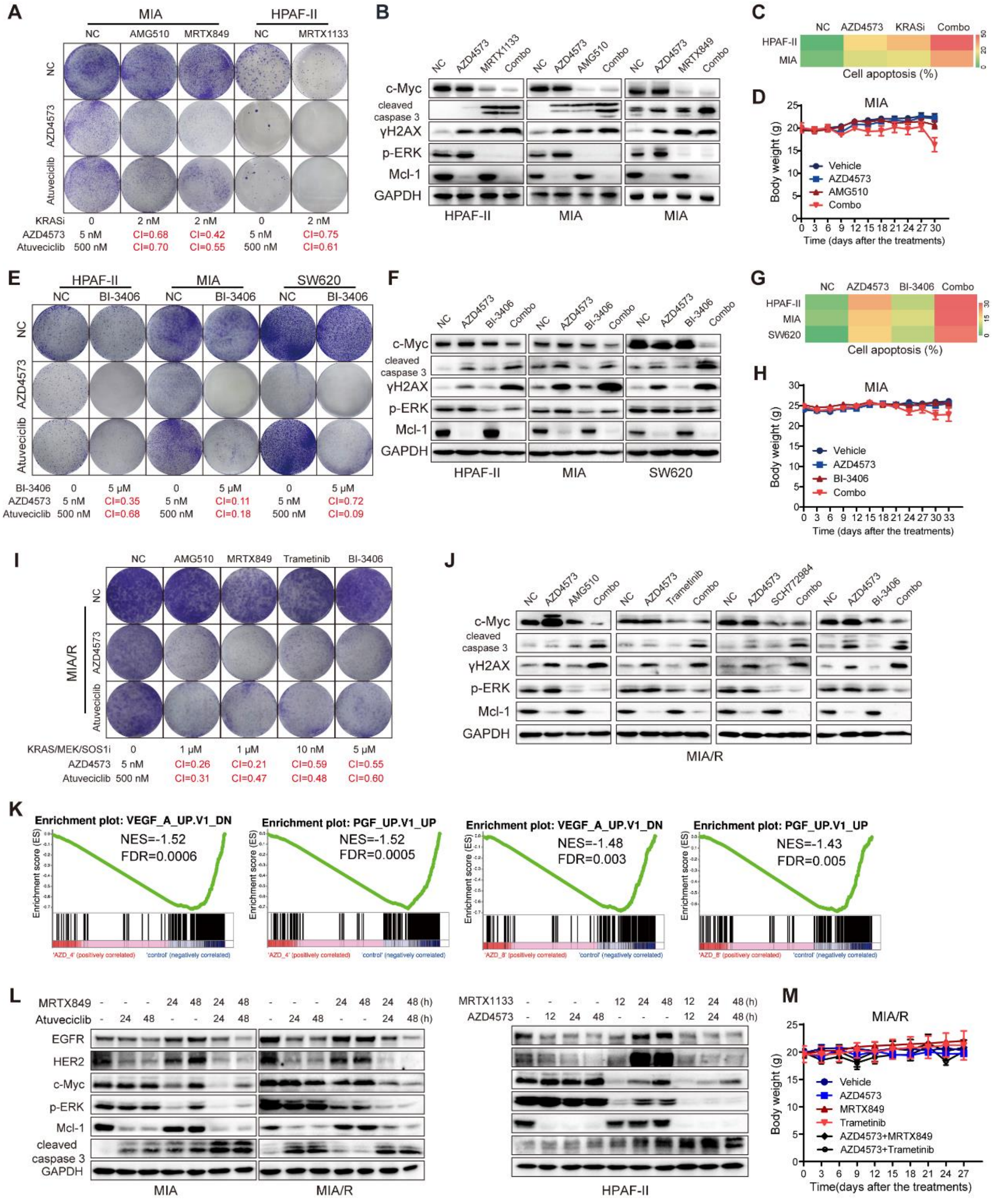

Figure S5 Combined inhibition of CDK9 and KRAS signaling diminishes ERK phosphorylation and abrogates feedback activation of RTKs and Mcl-1.

(A) Clonogenic assay of MIA and HPAF-II treated with control (DMSO), AZD4573 (5 nM)/Atuveciclib (500 nM), and KRASi (AMG510 or MRTX849, MRTX1133, 2 nM), alone or in combination. (B) Western blot analysis of cell lines HPAF-II and MIA treated with AZD4573 in 40 nM alone or combined with 100 nM KRASi for 24 h. (C) Heatmap showing the cell apoptosis in HPAF-II and MIA after treatment of AZD4573 (40 nM) or KRASi (MRTX1133 or AMG510, 100 nM), alone or in combination for 48 h (shown are mean values, n=3). (D) Mouse body weights of MIA xenograft models after the co-treatment with AZD4573 and AMG510. Data are the mean values of mouse body weight for each group (n = 5); error bar: SEM. (E) Clonogenic assay of MIA, HPAF-II and SW620 cells treated with control (DMSO), AZD4573 (5 nM)/Atuveciclib (500 nM), or BI-3406 (5 μM), alone or in combination. (F) Western blot analysis of cell lines HPAF-II, MIA and SW620 treated with AZD4573 (40 nM) alone or combined with BI-3406 (10 μM) for 24 h. (G) Heatmap showing the cell apoptosis after treatment of AZD4573 and BI-3406, alone or in combination for 48 h (shown are mean values, n=3). (H) Mouse body weights of MIA xenograft models after the co-treatment with AZD4573 and BI-3406. Data are the mean values of mouse body weight for each group (n = 5); error bar: SEM. (I) Clonogenic assay of MIA/R cells treated with control (DMSO), AZD4573 (5 nM)/Atuveciclib (500 nM), or AMG510 (1 μM)/MRTX849 (1 μM)/Trametinib (10 nM)/BI-3406 (5 μM), alone or in combination. (J) Western blot analysis of MIA/R cells treated with 40 nM AZD4573 alone or combined with AMG510 (100 nM)/Trametinib (100 nM)/SCH772984 (100 nM)/BI-3406 (10 μM) for 24 h. (K) GSEA plots of gene sets involved in VEGF and PGF signaling in HPAF-II cells treated with AZD4573 for 4 or 8 h. (L) Immunoblotting assays of the indicated protein levels in MIA, MIA/R and HPAF-II cells treated with Atuveciclib (1 μM) or AZD4573 (40 nM) in combination with 100 nM MRTX849 or MRTX1133 for indicated times. (M) Mouse body weights of MIA/R xenograft models after treatments with AZD4573 alone or in combination with MRTX849/Trametinib. Data are the mean values of mouse body weight for each group (n = 5); error bar: SEM.

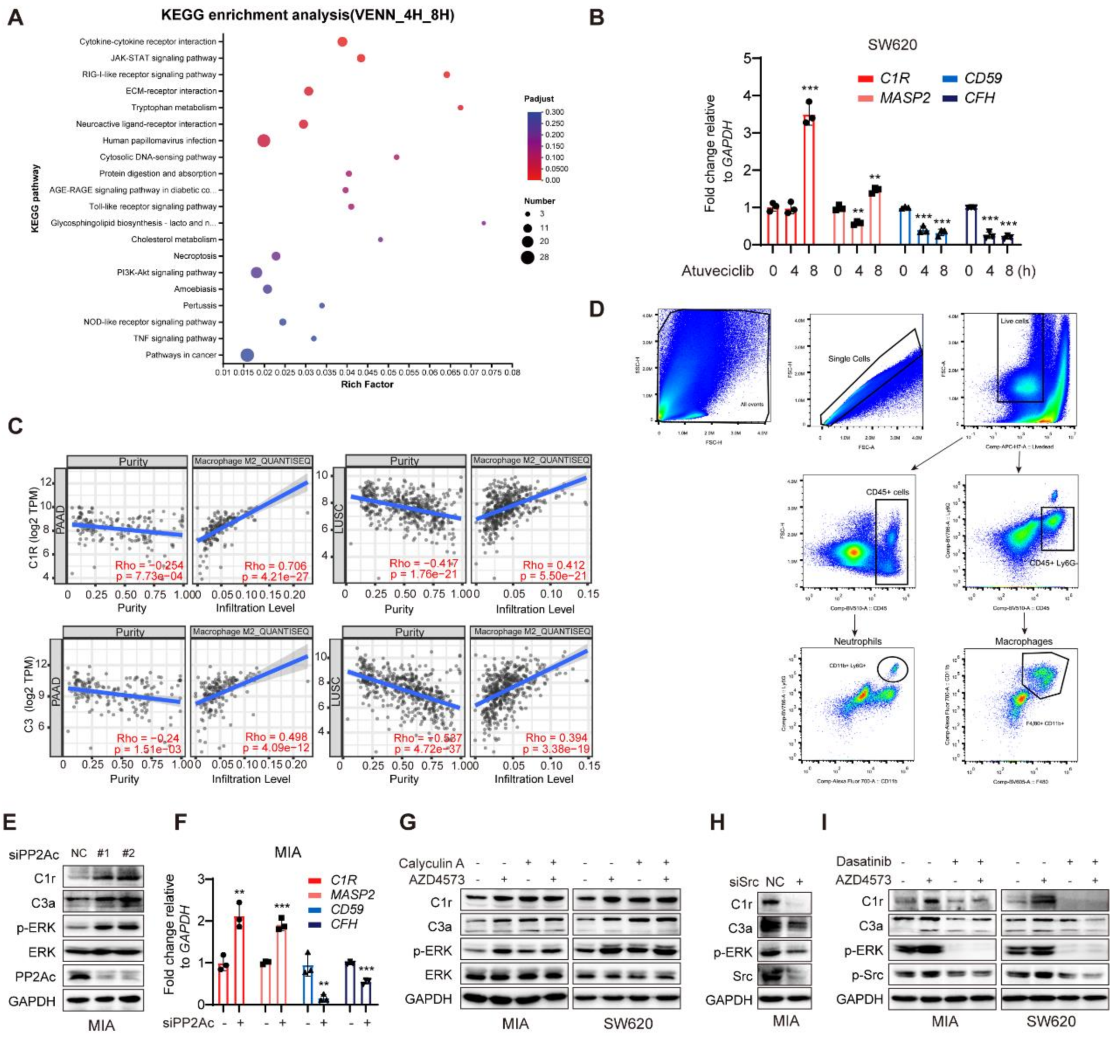

Figure S6 ERK-mediated C1r expression promotes complement activation and TME remodeling.

(A) KEGG analyses of the commonly deregulated AZD4573 (4 or 8 h) signature genes identified in HPAF-II cells. (B) Bar plots showing relative expression levels of complement genes after Atuveciclib treatment for 4 or 8 h in SW620 determined by RT-qPCR. (C) The expression of C1R and C3 have significant positive correlations with infiltrating levels of M2 macrophages in PAAD and LUSC via the TIMER database. (D) Gating strategies for flow cytometry analysis of immune cells from HPAF-II xenograft tumors. MIA cells were transfected with siRNAs targeting PP2Ac. The protein levels were determined by western blot analyses (E) and the mRNA levels of complement genes were determined by RT-qPCR (F). (G) Immunoblots for C1r and C3a in MIA and SW620 cells with AZD4573 (40 nM) plus Calyculin A (2 nM) treatment for 12 h. (H) Immunoblots for C1r and C3a in MIA cells with siNC or siSrc treatment. (I) MIA and SW620 cells were treated with AZD4573 (40 nM) plus Dasatinib (100 nM) treatment for 12 h. The protein levels

were determined by western blot analyses. For all statistical plots, the data are presented as mean ± SD (n = 3). ns. indicates no significance; *p < 0.05, **p < 0.01, ***p < 0.001 by two-tailed unpaired Student's *t* test.

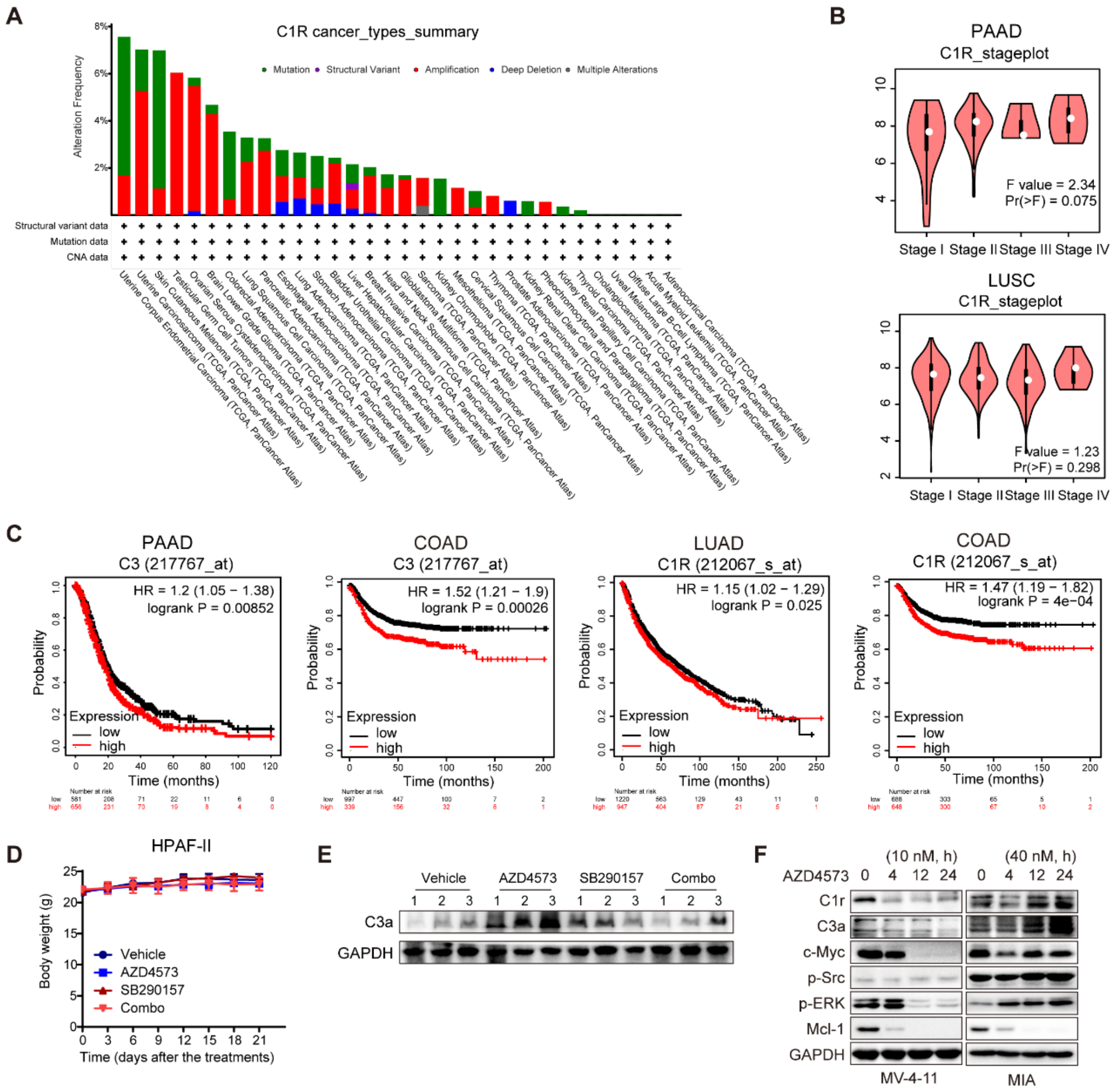

Figure S7 Prognostic significance of C1R and C3 in cancer patients.

(A) Cancer types summary of C1R alteration frequency via cBioPortal database. (B) Stage plot of C1R expression levels in PAAD and LUSC patients on GEPIA database. (C) C1R and C3 are highly expressed and correlates with poor prognosis in LUAD, PAAD, COAD and PAAD patients. (D) Mouse body weights of HPAF-II xenograft models after the co-treatment with AZD4573 and SB290157. Data are the mean values of mouse body weight for each experimental group (n = 5); error bar: SEM. (E) Tumors from mice treated as in (D) were harvested after the treatments and lysates were analyzed by immunoblotting for C3a protein. (F) Immunoblotting for the indicated proteins in MV-4-11 and MIA cells after AZD4573 treatments for the indicated times.

Supplemental tables

Table S1. Cell lines and culture conditions.

Cell lines	Culture condition	Source	Identifier
MIA	Dulbecco's Modified Eagle's Medium (DMEM) + 10% FBS + 2.5% horse serum	ATCC (Manassas, VA, USA)	CRM-CRL-1420
BxPC-3	RPMI-1640 + 10% FBS	ATCC	CRL-1687
HPAF-II	Eagle's Minimum Essential Medium (EMEM)+ 10% FBS	ATCC	CRL-1997
CFPAC-1	Iscove's Modified Dulbecco's Medium (IMDM) + 10% FBS	ATCC	CRL-1918
NCI-H358	RPMI-1640 + 10% FBS	ATCC	CRL-5807
NCI-H441	RPMI-1640 + 10% FBS	ATCC	HTB-174
NCI-H157	RPMI-1640 + 10% FBS	ATCC	CRL-5802
NCI-H460	RPMI-1640 + 10% FBS	ATCC	HTB-177
SW1573	Leibovitz's L-15 + 10% FBS	ATCC	CRL-2170
A549	F-12K + 10% FBS	ATCC	CRM-CCL-185
SW1463	Leibovitz's L-15 + 10% FBS	ATCC	CCL-234
Gp2d	DMEM + 10% FBS	European Collection of Authenticated Cell Cultures (ECACC)	95090714
SW620	Leibovitz's L-15 + 10% FBS	ATCC	CCL-227
LS174T	EMEM + 10% FBS	ATCC	CL-188
MV-4-11	IMDM + 10% FBS	ATCC	CRL-9591
293T	DMEM + 10% FBS	ATCC	CRL-3216
Ba/F3	RPMI-1640 + 10% FBS	German Collection of Microorganisms and Cell Cultures (DSMZ)	ACC 300

Table S2. Reagents and Tools table.

**Antibodies and reagents**	**Source**	**Identifier**
Antibodies		
pThr202/pTyr204 ERK1/2 H&M	Cell Signaling Technologies	Cat# 4370
ERK1/2 H&M	Cell Signaling Technologies	Cat# 4695
PP2Ac H&M	Santa Cruz	Cat# sc-80665
Phospho-PPP2CA/PPP2CB-Y307 H&M	ABclonal	Cat# AP0927
EGFR H&M	Cell Signaling Technologies	Cat# 4267
HER2 H&M	Cell Signaling Technologies	Cat# 2165
FGFR1 H&M	Cell Signaling Technologies	Cat# 9740
Histone H3 H&M	Cell Signaling Technologies	Cat# 4499
GAPDH H&M	Cell Signaling Technologies	Cat# 5174
Cleaved caspase-3 H	Cell Signaling Technologies	Cat# 9661
γH2AX H&M	Cell Signaling Technologies	Cat# 9718

c-Myc H&M	Cell Signaling Technologies	Cat# 18583
Phospho-c-Myc (Ser62)	Cell Signaling Technologies	Cat# 13748
Pol II-pSer2 H&M	Cell Signaling Technologies	Cat# 13499
Pol II-pSer5 H&M	Cell Signaling Technologies	Cat# 13523
Pol II CTD H&M	Cell Signaling Technologies	Cat# 2629
STAT3 H&M	Cell Signaling Technologies	Cat# 9139
Phospho-STAT3 (Tyr705) H&M	Cell Signaling Technologies	Cat# 9145
SAPK/JNK H&M	Cell Signaling Technologies	Cat# 9252
Phospho-SAPK/JNK H&M	Cell Signaling Technologies	Cat# 9251
Src H&M	Cell Signaling Technologies	Cat# 2109
Phospho-Src Family (Tyr416) H&M	Cell Signaling Technologies	Cat# 6943
p38 H&M	Cell Signaling Technologies	Cat# 8690
Phospho-p38 H&M	Cell Signaling Technologies	Cat# 4511
p38 H&M	Cell Signaling Technologies	Cat# 8690
Phospho-eIF2α (Ser51) H&M	Cell Signaling Technologies	Cat# 3398
CHOP H&M	Cell Signaling Technologies	Cat# 2895
LC3B H	Cell Signaling Technologies	Cat# 3868
Phospho-c-Jun (Ser73) H&M	Cell Signaling Technologies	Cat# 3270
c-Jun H&M	ABclonal	Cat# A0246
Mcl-1 H&M	Cell Signaling Technologies	Cat# 5453
NRF2 H&M	Cell Signaling Technologies	Cat# 12721
HIF-1α H&M	Cell Signaling Technologies	Cat# 36169
Myc-tag H&M	Cell Signaling Technologies	Cat# 14793
HA-tag H&M	Cell Signaling Technologies	Cat# 3724
GAPDH H&M	Cell Signaling Technologies	Cat# 4970
C1r H	Abcam	Cat# ab185212
C3 H&M	Abcam	Cat# ab200999
CD206 H&M	Abcam	Cat# ab64693
Ly6G M	Abcam	Cat# ab238132
CD31 H&M	Abcam	Cat# ab281583
Goat anti-Mouse IgG (H+L) Highly Cross-Adsorbed Secondary Antibody, Alexa Fluor™ 594	Thermo Fisher Scientific	Cat#: A-11032; RRID:
Goat anti-Rabbit IgG (H+L) Cross-Adsorbed Secondary Antibody, Alexa Fluor™ 488	Thermo Fisher Scientific	Cat# A-11008; RRID:
LIVE/DEAD™ Fixable Near-IR Dead Cell Stain Kit for 633 or 635nm excitation	Invitrogen	Cat# L34975
Purified Rat anti-mouse CD16/CD32(mouse BD Fc Block)	BD	Cat# 553142
BV510 anti-mouse CD45 antibody	BD	Cat# 563891
BV786 anti-mouse Ly6G antibody	BD	Cat# 740953
AF700 anti-mouse CD11b antibody	BD	Cat# 557960
Brilliant Violet 605™ anti-mouse F4/80 Antibody (BM8)	Biolegend	Cat# 123133
**Chemicals, peptides, and recombinant proteins**		

AZD4573	Selleck Chemicals	Cat# S8719
Atuveciclib	Selleck Chemicals	Cat# S8727
LDC000067	Selleck Chemicals	Cat# S7461
BAY1251152	Selleck Chemicals	Cat# S8730
THAL-SNS-032	Selleck Chemicals	Cat# S8979
Trametinib	MedChemExpress	Cat# HY-10999
SCH772984	MedChemExpress	Cat# HY-50846
AMG510	MedChemExpress	Cat#: HY-114277
MRTX849	MedChemExpress	Cat# HY-130149
MRTX1133	MedChemExpress	Cat# MRTX1133
MYCi975	Selleck Chemicals	Cat# S8906
Volasertib	MedChemExpress	Cat# HY-12137
S63845	MedChemExpress	Cat# HY-100741
BI-3406	MedChemExpress	Cat# HY-125817
STAT3-IN-3	MedChemExpress	Cat# HY-128588
ccs1477	MedChemExpress	Cat# HY-111784
MS275	MedChemExpress	Cat# HY-12163
NAC	MedChemExpress	Cat# HY-B0215
SB290157	MedChemExpress	Cat# HY-101502A
Dasatinib	MedChemExpress	Cat# HY-10181
DT-061	MedChemExpress	Cat# HY-112929
Calyculin A	MedChemExpress	Cat# HY-18983
**Reagents**		
Fetal bovine serum (FBS, Gibco)	Thermo Fisher Scientific	Cat#:10099141
Fetal bovine serum (FBS, Biosera)	Biosera	Cat#: FB-1058-500
Non-essential amino acids (NEAA)	Thermo Fisher Scientific	Cat#:11140050
Sodium pyruvate	Thermo Fisher Scientific	Cat#:11360070
Penicillin/streptomycin solution (100X)	Beyotime Biotechnology	Cat#: MA0110-1
4',6-diamidino-2-phenylindol (DAPI)	Thermo Fisher Scientific	Cat#: D1306
Lipofectamine RNAiMAX	Thermo Fisher Scientific	Cat#: 13778150
Lipofectamine 2000	Thermo Fisher Scientific	Cat#: 11668019
TurboFectin 8.0	OriGene	Cat#: TF81001
RIPA lysis buffer	Thermo Fisher Scientific	Cat#: 89900
Dimethylsulfoxide (DMSO)	Sigma-Aldrich	Cat#: BML-KI597-0400
Thiazolyl Blue (MTT)	Sigma-Aldrich	Cat#: M5655
Dithiothreitol (DTT)	Sigma-Aldrich	Cat#: 43815
Sulforhodamine B (SRB)	Sigma-Aldrich	Cat#: 341738
Tween 80	Sigma-Aldrich	Cat#: P4780
Triton-X100	Sigma-Aldrich	Cat#: X100
Bovine serum albumin (BSA)	Sigma-Aldrich	Cat#: B2064
Normal goat serum	JACKSON	Cat#: 005-000-121
Normal Rabbit IgG	CST	Cat#: 2729S
Protein A/G magnetic beads	Thermo Fisher Scientific	Cat#: 88802

Goat anti-Rabbit IgG (H+L) Secondary Antibody, HRP	Invitrogen	Cat#: 65-6120
Protease inhibitor cocktail	Roche	Cat#: 04693159001
Phosphatase inhibitor cocktail	Roche	Cat#: 04906837001
RNase A	Beyotime Biotechnology	Cat#: ST576
Propidium iodide (PI)	Beyotime Biotechnology	Cat#: ST511
Phosphate buffer saline (PBS)	Beyotime Biotechnology	Cat#: C0221A
4% paraformaldehyde	Beyotime Biotechnology	Cat#: 10455491
NP-40 lysis buffer	Beyotime Biotechnology	Cat#: ST366
Sodium dodecyl sulfate (SDS)	Biosharp	Cat#: BS028B
Proteinase K	ABclonal	Cat#: RP02503LQ
Human CDK9/Cyclin T1, His Tag Recombinant Protein	Thermo Fisher Scientific	Cat#: PV4131
ATP solution (100 mM)	Thermo Fisher Scientific	Cat#: R0441
Threonine phosphopeptide	Sigma-Aldrich	Cat#: #12-219
Phosphate	Bio Assay Systems	Cat#: DIPI-500
Malachite green solution	Sigma-Aldrich	Cat#: 38978-100ML-F
2X Saline Sodium Citrate Buffer	MACKLIN	Cat#: X861541
**Recombinant DNA**		
pDEST40-2XHA-Src-WT	Addgene	Cat#140294
pDEST40-2XHA-Src-KD	Addgene	Cat#140312
pDEST40-2XHA-Src-K298M-Y419F-Y530F (3M)	Addgene	Cat#140320
pCDNA3-1-MYC-HISA-PP2Ac	This paper	N/A
pCDNA3-1-MYC-HISA-PP2Ac T281A	This paper	N/A
pCDNA3-1-MYC-HISA-PP2Ac Y284F	This paper	N/A
**Critical commercial assays**		
BCA Protein Assay Kit	Thermo Fisher Scientific	Cat#: 23227
PARIS RNA Purification Kit	Thermo Fisher Scientific	Cat#: AM1921
PrimeScript™ RT Master Mix (Perfect Real Time)	Takara	Cat#: RR036A
SYBR Green PCR Master Mix	Vazyme	Cat#: Q111-02
Annexin V-FITC/PI double staining apoptosis kit	BD biosciences	Cat#: 556547
Human IL-10 ELISA Kit	RayBiotech	Cat#: P22301
Human TGF-β ELISA Kit	RayBiotech	Cat#: P01137
Human IL8 ELISA Kit	Abcam	Cat#: ab214030
SimpleChIP® Plus Enzymatic Chromatin IP Kit (Magnetic Beads)	CST	Cat#: 9005
NE-PER Extraction reagents	Thermo Fisher	Cat#: 78833
Human Cytokine Array C5	RayBiotech	Cat#: AAH-CYT-5
**Software and Algorithms**		
GraphPad Prism software 8.0	N/A	https://www.graphpad.com/
GSEA	N/A	http://software.broadinstitute.org/gsea/index.jsp
DAVID	N/A	http://david.abcc.ncifcrf.gov/
FlowJo (version 10.7.2)	BD Biosciences	https://www.flowjo.com/solutions/flowjo/downloads

Image J	Schneider et al.[1]	https://imagej.nih.gov/ij/
sgRNA design tool	Benchling	https://benchling.com
CalcuSyn software	Biosoft	https://calcusyn.software.informer.com/download/

Table S3. siRNA sequences in RNA interference.

Gene	siRNA sense (5'-3')
CDK9 #1	GCAAGGGUAGUAUAUACCUGGUGTT
CDK9 #2	CAUACGCACUGGACCUCAUCGACAA
KRAS #1	CUAUGGUCCUAGUAGGAAA
KRAS #2	UUUCCUACUAGGACCAUAG
ERK1	GAGAUGUCUACAUUGUGCATT
ERK2	GCGCTTCAGACATGAGAACAT
c-Myc #1	GUGCAGCCGUAUUUCUACUTT
c-Myc #2	GAACACACAACGUCUUGGATT
PP2Ac #1	CAAACAATCAUUGGAGCUUAA
PP2Ac #2	ACACCUCGUGAAUACAAUUUA
Src #1	CUCGGCUCAUUGAAGACAATT
Src #2	GAAGAAGCUGAGGCAUGAGAA
siNC	UUCUCCGAACGUGUCACGU

Table S4. Sequences in RT-qPCR and CHIP-qPCR.

Gene	Forward (5'-3')	Reverse (5'-3')
MYC	GGCTCCTGGCAAAAGGTCA	CTGCGTAGTTGTGCTGATGT
JUNB	CACCAAGTGCCGGAAGCGGA	AGGGGCAGGGGACGTTCAGA
ERG1	CACCTGACCGCAGAGTCTT	GCGGCCAGTATAGGTGATGG
FOS	CACTCCAAGCGGAGACAGAC	GAGCTGCCAGGATGAACTCT
MCL1	GCGACTTTTGGCCACCG	TGATGTCCAGTTTCCGAAGCA
EGFR	GGTGAGTGGCTTGTCTGGAA	CCTTACGCCCTTCACTGTGT
ERBB2	GTTCCCGGATTTTTGTGGGC	CATGGGGAAGCAATCACCCT
C1R	CAGAGAGGAGAATGCCCAGT	ACGAGCTTCACCCTGTATCC
MASP2	GATGATCTACCCAGTGGCCG	CTCCTTTGGAGCTCGTCCAG
CD59	CTGGAAGAGGATCTTGGGCG	AGGACAGACCCTCCTTGGAT
CFH	TCCGTGTCAAAACATGTTCCA	GGGTTGAGCTGACCATCCAT
CXCL8	CTCCAAACCTTTCCACCCCA	TTCTCAGCCCTCTTCAAAAACT
MTRES1	CAGTCTTCGATTATAAGCGCCAT	ATGAAGGTGTCCCTCGGAGA
MTRF1	CACCTAGCCGAGGAGAGACT	CTTCCTTTCCGCGGTCTTCT
MTO1	AGTGATTCAGCCAGGCTACG	TGCCATTGATCTGTCCAGCA
ATP5A	CACGCAGGTGTTCTTCAACG	AGTATTTGGAGGTGGTGCCG
ACTB	GAGAAAATCTGGCACCACACC	ATACCCCTCGTAGATGGGCAC
GAPDH	GACATCAAGAAGGGGTGAA	TGTCATACCAGGAAATGAGC
mTnf	CCCTCACACTCACAAACCAC	ACAAGGTACAACCCATCGGC
mIl1b	TGCCACCTTTTGACAGTGATG	TGATGTGCTGCTGCGAGATT

mInos	CTGGGAGCGCTCTAGTGAAG	CTCTCCACTGCCCCAGTTTT
mIfng	TCAAGTGGCATAGATGTGGAAGAA	TGGCTCTGCAGGATTTTCATG
mIl12a	GGAAGCACGGCAGCAGAATA	AACTTGAGGGAGAAGTAGGAATGG
mFizz1	CCCTTCTCATCTGCATCTCC	CTGGATTGGCAAGAAGTTCC
mArg1	AGAGATTATCGGAGCGCCTT	TTTTTCCAGCAGACCAGCTT
mIl10	GGTTGCCAAGCCTTATCGGA	ACCTGCTCCACTGCCTTGCT
mRpl13	TGATTGGCGTTTGAGATTGGC	AATCCTTGTGGAAGTGGGGC
MYC distal	ATTAACCGGGTGTGGTGGTGC (-1997 to -1977)	TCTCCTTCTGTCACCCAGGCT (-1869 to -1849)
MYC proximal	TTTTAGGAAGTCCGGTCCCGC (-452 to -432)	AGACAAATCCCCTTTGCGCCCT (-335 to -314)
MYC TSS	ACTCCCCCCAACAAATGCAA (-21 to -2)	GGCATAAGGAGGAAAACGATGC (+71 to +92)
MYC gene body	ATCCCACGCTCTGAACGC (+121 to +138)	TAAATCATCGCAGGCGGAAC (+290 to +309)
JUNB distal	TGCCTAGCCCACAGTAAGAGC (-3121 to -3101)	TGTCACGCAGACAGTGAGACT (-3034 to -3014)
JUNB proximal	AAACCCCTCACTCATGTGC (-276 to -258)	TCACTGTCAGGAAGCGCGTGT (-154 to -134)
JUNB TSS	GGCTGGGACCTTGAGAGC (-3 to +15)	GTGCGCAAAAGCCCTGTC (+116 to +133)
JUNB gene body	ACGACGACTCATACACAGCTA (+1317 to +1336)	TTTGAGACTCCGGTAGGGGT (+1347 to +1366)
FOS distal	GATCTAGTTGTGAATGGCAGTCATG (-2225 to -2201)	TCAAGCTTTGAATTCCTGAGTCTG (-2198 to -2175)
FOS proximal	AGCAGTTCCCGTCAATCCCT (-352 to -333)	TGTCTCAGAGGTCTCGTGG (-222 to -204)
FOS TSS	TGAGCCCGTGACGTTTAC (-73 to -56)	TGCAGATGCGGTTGGAG (-5 to +13)
FOS gene body	CGATGATGTTCTCGGGCTTCAA (+1070 to +1089)	TGCGGGTGAGTGGTAGTAAGAGA (+1105 to +1122)
C1R distal	AGCACTGGACTGAAGTTCTTGC (-1978 to -1957)	ATTACAGGCGTGAGCCACT (-1857 to -1839)
C1R proximal	ATCTGGTGCTTTTCCTTCTCCC (-375 to -354)	TTGGAGGGTTTCTCCAGCAGG (-251 to -231)
C1R TSS	GGGAAACAAGCAGAACAGCCTA (-26 to -5)	GGCTTTCTTCATCAACTCTCC (+77 to +79)
C1R gene body	AGAGACTGAGACATCTGGGCCT (+836 to +857)	TGTTTGTGGTGCCACCTGCTG (+925 to +945)

Table S5. A table showing some of the CDK9 interacting partners with high scores and high LFQ intensities in the LC-MS/MS data.

Protein names	Gene names	Score	Unique peptides	Mean LFQ intensity NC	Mean LFQ intensity 8 h	Fold change (8h/NC)
Heat shock protein HSP 90-alpha	HSP90A	269.3	22	1.53E+09	1.19E+09	0.78
Heat shock cognate 71 kDa protein	HSPA8	262.94	22	3.75E+09	3.09E+09	0.82
Eukaryotic initiation factor 4A-I	EIF4A1	232	18	1.22E+09	1.67E+09	1.37
Splicing factor proline and glutamine rich	SFPQ	220.65	24	1.56E+09	2.71E+09	1.74
DNA topoisomerase 1	TOP1	209.72	28	1.90E+09	2.49E+09	1.31
RNA-binding protein EWS	EWSR1	197.77	2	2.70E+09	6.59E+09	2.44
RNA-binding protein 14	RBM14	183.63	24	1.28E+09	1.23E+09	0.96
Elongation factor 1-alpha 1	EF1A1;EEF1A1P	183.11	13	4.02E+09	4.35E+09	1.08
Heat shock 70 kDa protein 1B;1A	HSPA1B;HSPA1A	179.22	9	1.28E+09	9.60E+08	0.75
RNA-binding protein FUS	FUS	175.25	9	2.51E+09	2.48E+09	0.99
ATP-dependent RNA helicase DDX3X; DDX3Y	DDX3X;DDX3Y	174.62	21	1.09E+09	8.47E+08	0.78
14-3-3 protein zeta/delta	YWHAZ	162.9	7	7.80E+08	1.18E+09	1.51
Nucleolar RNA helicase 2	DDX21	159.23	28	1.29E+09	1.13E+09	0.88
AP-2 complex subunit alpha-1	AP2A1	149.24	22	1.63E+09	1.10E+09	0.67
Interleukin enhancer-binding factor 3	ILF3	142.63	23	8.33E+08	8.94E+08	1.07
Catenin alpha-1	CTNNA1	132.77	19	4.47E+08	4.44E+08	0.99
Bromodomain containing 4	BRD4	130.42	13	6.04E+08	7.23E+08	1.20
Upstream binding transcription factor 1	UBTF	127.88	12	4.01E+08	3.77E+08	0.94
Serine/threonine-protein phosphatase 2A catalytic subunit alpha	PPP2CA; PPP2CB	125.23	7	1.19E+08	2.81E+08	2.36
Elongation factor 2	EEF2	119.72	25	9.69E+08	1.42E+09	1.47
Probable ATP-dependent RNA helicase DDX5	DDX5	110.68	16	1.84E+09	1.55E+09	0.84
AP-2 complex subunit beta	AP2B1	103.34	16	1.19E+09	7.83E+08	0.66
DBIRD complex subunit ZNF326	ZNF326	103.33	9	9.25E+07	1.74E+08	1.88
Serine/threonine-protein phosphatase 2A 55 kDa regulatory B alpha	PPP2R2A	101.39	6	6.75E+07	8.87E+07	1.31
Serine/threonine-protein phosphatase PP1-beta catalytic subunit	PPP1CB	94.72	4	3.87E+08	4.92E+08	1.27
FACT complex subunit SPT16	SUPT16H	94.353	19	5.87E+08	7.53E+08	1.28
Pre-mRNA-splicing factor DHX15	DHX15	87.304	23	7.68E+08	8.38E+08	1.09
Regulator of chromosome condensation	RCC1	87.185	15	3.11E+08	5.81E+08	1.87
Serine/threonine-protein phosphatase PP1-alpha catalytic subunit	PPP1CA	85.7318	3	7.70E+07	1.29E+08	1.68
AP-2 complex subunit mu	AP2M1	81.12	8	2.21E+08	1.73E+08	0.78
Signal transducer and activator of transcription 3	STAT3	76.791	16	2.91E+08	3.79E+08	1.30
Transducin beta-like protein 3	TBL3	76.656	18	3.11E+08	2.11E+08	0.68

Mitogen-activated protein kinase 3; Mitogen-activated protein kinase	MAPK3	76.46	2	9.03E+06	9.99E+06	1.11
Catenin beta-1	CTNNB1	65.602	15	2.63E+08	2.62E+08	1.00
Elongation factor 1-gamma	EEF1G	65.088	11	3.61E+08	4.50E+08	1.25
WD repeat-containing protein 36	WDR36	61.395	14	1.55E+08	9.79E+07	0.63
AP-2 complex subunit alpha-2	AP2A2	61.198	7	4.05E+08	3.55E+08	0.88
E1A binding protein p300	EP300	57.454	5	8.85E+07	1.42E+08	1.60
Proto-oncogene tyrosine-protein kinase Src	SRC	48..48	4	2.13E+07	2.50E+07	1.17
WD repeat-containing protein 1	WDR1	45.138	13	2.49E+08	2.58E+08	1.04
Serine/threonine-protein phosphatase 2A 65 kDa regulatory A alpha	PPP2R1A	40.272	8	1.46E+08	1.55E+08	1.06
Serine/threonine-protein phosphatase PGAM5, mitochondria	PGAM5	35.819	14	1.63E+08	3.29E+08	2.02
Integrator complex subunit 3	INTS3	33.6336	2	5.65E+07	9.75E+07	1.72
Elongation factor 1-delta	EEF1D	30.474	3	3.84E+07	7.20E+07	1.87
Histone deacetylase 1; Histone deacetylase 2	HDAC1; HDAC2	24.416	5	1.25E+08	1.62E+08	1.30

Supplementary materials and methods

Cell lines

The human cancer cell lines BxPC-3, MIA PaCa-2, HPAF-II, CFPAC-1, NCI-H358, NCI-H441, NCI-H157, NCI-H460, SW1573, A549, SW1463, SW620, LS174T, MV-4-11 and HEK293T were obtained from American Type Culture Collection (ATCC, Manassas, VA, USA). Gp2d was obtained from European Collection of Authenticated Cell Cultures (ECACC, UK) and Ba/F3 was obtained from German Collection of Microorganisms and Cell Cultures (DSMZ, Germany). These cells were cultivated in a medium supplemented with 10% fetal bovine serum (Gibco, NY, USA or Biosera, Grosseron, France) and 1% penicillin-streptomycin (Beyotime Biotechnology, Shanghai, China) at 37°C with 5% CO_2 in a humidified incubator (Thermo Fisher Scientific, Waltham, MA, USA). More specific information is provided in Table S1. Cell lines were authenticated by short tandem repeat (STR) DNA profiling via the cell line bank from which they were obtained. Cell lines were routinely tested for Mycoplasma using the MycoBlue Mycoplasma Detector Kit (Vazyme, D101-01).

SiRNA, plasmids, and transfection

Lentiviral vectors encoding KRAS-mutant genes were constructed by our laboratory. The related plasmids were transfected into the cells using Lipofectamine® 2000 (Invitrogen, CA, USA). Stable Ba/F3 cell lines were selected using neomycin (Beyotime, T1450, 600 μg/mL) for 7-10 days.

siRNAs were synthesized by Tsingke (Shanghai, China) and transfected into cells using Lipofectamine RNAiMAX transfection reagent (Thermo Fisher Scientific). For proliferation analysis, cells were incubated for 24 h post-siRNA transfection to ensure knockdown prior to the addition of drugs. Transfection efficiency was confirmed using RT-qPCR and western blotting. Plasmids used were obtained from Addgene and are listed in Table S2. The siRNA sequences used are listed in Table S3.

Cell viability assays and drug combination analysis

Briefly, 1,000-8,000 cells per well were seeded in 96-well plates. After 12 h, cells were treated with DMSO or serial dilutions of the indicated drugs for 3 days and then assessed by the sulforhodamine B (SRB) or methylthiazolyldiphenyl-tetrazolium bromide (MTT) assays as described previously [2]. The optical density (OD) values of luminescence at 540 nM (SRB) or 490 nM (MTT) were recorded on a multimode reader (TECAN, Spark). The average IC_{50} values (mean ± SD) from three independent tests were generated using GraphPad Prism 8 (RRID:SCR_002798).

The combination index (CI) was calculated by the CompuSyn software following the Chou–Talalay equation.[3] CI <1, CI = 1, and CI >1 represent synergism, additive effect, and antagonism, respectively.

Crystal violet colony formation assays

Cells were seeded at the proper density per well in 6-well or 12-well plates, with three technical replicates per condition. Media with or without the indicated drugs was changed every 3 days for 7-10 days. Cells were washed with PBS, fixed in methanol at 4°C for 15 min, stained with 0.5% crystal violet for 20 min, and quantified using ImageJ.

Quantitative real–time PCR (RT-qPCR)

Total RNA was extracted from tissue specimens and cells using the TRIzol method (Invitrogen). A total of 5 μg of RNA was reverse transcribed and amplified using PrimeScript™ RT Master Mix (TaKaRa, RR036A) and SYBR Green PCR Master Mix (Vazyme) on an Applied Biosystems 7500HT Fast real-time PCR system (Thermo Fisher Scientific) according to the manufacturer's instructions. Gene expression values were calculated as $2^{-\Delta\Delta Ct}$. The housekeeping genes *GAPDH* in human cells or *Rpl13* in mouse cells were used to normalize the relative mRNA levels of target genes. The primers used are listed in Table S4.

Western blotting

Total protein was extracted from the tumor cells and tumor tissues using RIPA buffer, and a bicinchoninic acid (BCA) assay kit (Thermo Fisher Scientific) was used for concentration measurement. After quantification, protein samples were separated on sodium dodecyl sulfate-polyacrylamide gels (SDS-PAGE) and transferred to polyvinylidene difluoride (PVDF) or nitrocellulose (NC) filter membranes (Roche, Shanghai, China). After blocking in 3% BSA TBST buffer for 1 h, the membranes were incubated with primary antibodies at 4°C overnight, followed by 3 washes in TBST and incubation with horseradish peroxidase (HRP)-conjugated secondary antibody at room temperature for 1 h. After 3 washes, Pierce ECL detection reagent (Thermo Fisher Scientific) was used to visualize the protein bands using a chemiluminescent gel imaging system (Tanon4600, Shanghai), with GAPDH serving as the endogenous control. The antibodies used are listed in Table S1.

RNA-sequencing and data analysis

Total RNA was extracted from HPAF-II control-, or AZD4573-treated cells for 4 or 8 h using TRIzol® Reagent (Invitrogen) and RNA quality was determined by 2100 Bioanalyser (Agilent) and quantified using an ND-2000 (NanoDrop Technologies). An RNA-seq transcriptome library was generated with a TruSeqTM RNA sample preparation kit from Illumina (San Diego, CA) using 1 μg of total RNA and sequenced by Majorbio Company (Shanghai, China). FASTQ files were aligned to the human reference genome (version: GRCH38.p13; source: http://asia.ensembl.org/Homo_sapiens/Info/Index). Clean reads were separately aligned to the reference genome in orientation mode using HISAT2 (http://ccb.jhu.edu/software/hisat2/index.shtml) software and converted into binary alignment map (BAM) files. The value of the exon model per kilobase base fragment per million gene location fragments (FPKM) for each identified gene was calculated using Stringtie's default parameters. Subsequently, DESeq2 was used to identify significantly differentially expressed genes (DEGs) based on the following criteria: multiple variations and corrected p value were >2 and <0.05, respectively. All the RNA-seq data were normalized to time-matched DMSO samples for each time point. GSEA was then performed on the outputs of the ''Hallmark'' and "GO biological processes signature" gene sets in the Molecular Signatures Database (MSigDB v7.0,

http://software.broadinstitute.org/gsea/downloads.jsp).

Label-free proteomics analysis

Label-free quantification was used to compare protein abundance between the AZD4573-treated and control groups. Briefly, HPAF-II cells were treated with or without 40 nM AZD4573 for 8 h and lysed with cold IP buffer. Quantitative protein lysates were immunoprecipitated with primary anti-CDK9 antibody and protein A/G magnetic beads (Santa Cruz Biotechnology). After binding, the beads were then washed with IP lysis buffer three times and PBS three times on ice, followed by elution using SDS lysis buffer. The immunoprecipitated proteins were separated by SDS–polyacrylamide gel electrophoresis and the bands were extracted from the gel. For the mass spectrometry assay, the immunoprecipitates were incubated 10 mM DTT at 35°C for 30 min, followed by the addition of 20 mM iodoacetamide in the dark at 35°C for another 30 minutes. Then, trypsin digestion was carried out overnight at 37°C with rotation. The labeled peptides were dried using a vacuum centrifuge and subjected to analysis by liquid chromatography-tandem mass spectrometry (LC-MS/MS).

Subcellular fractionation

Nuclei and cell cytosol were isolated using the NE-PER extraction reagents from Thermo Fisher as described previously [4]. Briefly, cells after treatment were washed with cold PBS, centrifuged at 500 × g for 5 min, and then suspended in 200 µl of cytoplasmic extraction reagent I. After incubating on ice for 10 min, the suspension was mixed with 11 µl of cytoplasmic extraction reagent II and centrifuged at 16,000 × g for 5 min. The resulting supernatant contained the cytoplasmic protein while the insoluble pellet fraction was lysed in nuclear extraction reagent and centrifuged to obtain the nuclear extract.

Flow cytometry for ROS and cell apoptosis analysis

Intracellular and mitochondrial ROS were measured using 2',7'-dichlorofluorescin diacetate (H_2-DCFDA) and MitoSOX Red, respectively, following the manufacturer's instructions, respectively. Cells that received CDK9i or combination treatments were loaded with H_2-DCFDA (10 µM) or MitoSOX Red (5 µM) for 30 min, followed by flow cytometric analysis using CytoFLEX (Beckman, Pasadena, California, USA).

For cell apoptosis analysis, cells were incubated with the indicated drugs for 48 h and then

analyzed by Annexin V-FITC/PI double staining-based flow cytometry. The proportion of apoptotic cells was analyzed by the FlowJo software.

Oxygen consumption measurements

To analyze mitochondrial respiration, a Seahorse XF96 Analyzer (Seahorse Biosciences, USA) was used to measure the oxygen consumption rate (OCR) in the treated cells. The cells were cultured in a 96 well XF96 plate at a density of 1 × 10^4 cells/well and incubated overnight. Cartridge plates for metabolic stress injections were hydrated for 24 h at 37 °C without CO_2 in calibrant solution. One hour prior to the assay, the medium in the XF96 plate was replaced by Seahorse Assay Medium (supplemented with 1% FBS, 2 mM L-glutamine, 1 mM HEPES, and 25 mM glucose) and the assay was performed according to the manufacture's procedure. The compounds were injected sequentially as follows: 1 mM oligomycin; 0.5 mM FCCP; 0.5 mM rotenone (from Seahorse XF Cell Mito Stress Test Kit, Seahorse Bioscience). The protocol and algorithm for OCR responses were analyzed using Wave 2.4 software (Seahorse Bioscience). OCR was normalized by total cellular protein concentrations.

Chromatin immunoprecipitation (ChIP) assay

After the treatments, approximately 1 × 10^7 cells were crosslinked with 1% formaldehyde in PBS at 25°C for 10 min. The reaction was terminated by the addition of 0.125 M glycine, and the cells were lysed with lysis buffer on ice. Chromatin DNA was ultrasonicated (15 cycles: 30 sec pulses, 30 sec cooling) to obtain a fragment of approximately 500 bp and then incubated with an anti-CDK9, anti-PP2Ac, anti-ERK or anti-Pol II antibodies. The chromatin bound to these proteins was precipitated using protein A/G magnetic beads (Santa Cruz Biotechnology, Dallas, Texas, USA). Following decrosslinking, the precipitated DNA was purified and analyzed using RT-qPCR to identify putative protein binding sites in the *JUNB, FOS, MYC* or *C1R* locus regions. The fold enrichment of binding relative to the input was calculated. IgG and random primers that were unable to specifically bind the indicated gene loci regions (off target), were utilized as negative controls. The primers used for CHIP-qPCR are listed in Table S4.

Coimmunoprecipitation (Co-IP) assay

The cells were successfully transfected with the desired plasmid and lysed with an ice-cold NP-40 lysis buffer. Next, the cell lysates containing the proteins were incubated with the indicated

antibodies or anti-tags overnight at 4°C and precipitated with protein A/G magnetic beads (Santa Cruz Biotechnology). Subsequently, the beads were washed, resuspended in SDS gel-loading buffer, and subjected to western blot analysis.

Expression of recombinant 6His-PP2Ac protein

The cDNA of human PP2Ac was amplified from plasmid pCDNA3-1-MYC-HISA-PP2Ac and inserted into plasmid pET-28a (+) through homologous recombination using ClonExpress Ultra One Step Cloning Kit (Vazyme, #C117). 6His-PP2Ac protein was expressed in BL21 (DE3) cells, lysed using a FB-110X high pressure cell crusher at 1000 psi (Litu, Shanghai, China) in binding buffer (50 mM Tris-HCL pH=8.0, 500 mM NaCl, 1×protease inhibitor cocktail, 10 mM β-mercaptoethanol), and immobilized onto Ni-NTA agarose resin (Yeasen, Shanghai, China). The resin was then washed 3 times for 5 min each with wash buffer (50 mM Tris-HCL pH=8.0, 500 mM NaCl, 10 mM β-mercaptoethanol, 30 mM imidazole). Subsequently, the proteins were eluted using 10 mL of elution buffer (50 mM Tris-HCL pH=8.0, 500 mM NaCl, 10 mM β-mercaptoethanol, 300 mM imidazole), with 1 mL collected fractions. Finally, the protein content was determined by SDS PAGE.

Kinase and phosphatase assays

To detect CDK9-dependent phosphorylation, recombinant CDK9/cyclin T1 complex (100 ng) was incubated with 500 ng of 6His-PP2Ac in kinase reaction buffer (25 mM HEPES, pH 7.4, 10 mM NaCl, 10 mM $MgCl_2$, 1 mM DTT and 10% glycerol) with or without ATP (1 mM) at 25°C for 30 min The reactions were boiled with 2×SDS buffer and subjected to SDS–PAGE for immunoblotting.

To measure protein phosphatase activity, the PP2Ac protein was immunoprecipitated from cells ectopically expressing the empty vector, Src-WT, Src-3M or Src-KD. A total of 10 mg of cell lysates was diluted in phosphatase assay buffer (50 mM Tris/HCl, pH 7.0; 100 mM $CaCl_2$) and incubated with threonine phosphopeptide (K-R-pT-I-R-R, #12-219, Merck) for 5 min at 30°C. Subsequently, malachite green solution (#38978-100ML-F, Sigma–Aldrich) was added, and the absorption was measured at different time points at a wavelength of 620 nm. The phosphatase activity in cells expressing the empty vector was determined to be 100%. A 40 mM phosphate (#DIPI-500, Bio Assay Systems) serial dilution was utilized as the assay standard.

Cell immunofluorescence (IF)

Cells cultured on coverslips were fixed with 4% paraformaldehyde for 15 min at RT after the treatments, permeabilized with PBS containing 0.1% Triton X-100 for 8 min and blocked for 30 min with PBS containing 1% BSA (Sigma-Aldrich). After incubation with primary antibodies overnight at 4°C, the samples were then washed and incubated with a goat anti-mouse Alexa 633 antibody and a goat anti-rabbit Alexa 488 antibody for 1 h at room temperature. For DNA detection, 4',6-diamidino-2-phenylindole (DAPI) (300 nM, Thermo Fisher Scientific) was used. The samples were sealed with the antifade mounting medium (Beyotime Biotechnology) and analyzed under a Leica LMS710 laser-scanning confocal microscope (Leica Microsystems, Germany).

Dissociation of tumor tissue and FACS analysis

Excised mouse tumor tissues were minced into smaller pieces (1-2 mm^3). Dissociation enzyme mixture was added and digested simultaneously using a Miltenyi gentle MACSTM Octo Dissociator. The digestion of the dissociated cells was stopped by the addition of 10 mL RPMI 1640 medium (with 10% FBS) after which the cells were passed through a 70 μm cell strainer and washed once with DPBS. A total of 1.2×10^6 cells were transferred to a 96-V bottom plate and gently stained with 100 μL of Live/Dead-APC-H7 (1:1000 in DPBS) for 10 minutes at room temperature. Then, the cells were stained with 30 μL of mouse Fc block (1:50 in staining buffer) for 10 min at room temperature. Next, an antibody mixture (BV510 anti-mouse CD45, BV786 anti-mouse Ly6G, AF700 anti-mouse CD11b and Brilliant Violet 605™ anti-mouse F4/80) was added and incubated for 30 minutes at 4°C in the dark. The cells were washed and resuspended in staining buffer. The antibodies used are listed in Table S1. Samples were acquired by a Cytek® Aurora flow cytometer and assessed using FlowJo software (Tree Star, CA, USA).

Tumor immunofluorescence (IF) staining

Briefly, tumor tissues were dewaxed and hydrated with xylene and a series of ethanol solutions (70%, 95%, and 100%), respectively. Endogenous peroxidase was inactivated using 0.3% hydrogen peroxide. The slides were incubated with the relevant primary antibodies overnight at 4°C and subsequently with fluorophore-conjugated secondary antibodies for 1 h. Labeling was completed by washing with 70% formamide, 10 mM Tris-HCl, and PBS, staining with DAPI, and sealing the slides for observation. Finally, images were captured and processed using a fluorescence microscope

(Tissue FAXS Plus Basic, TissueGnostics GmbH, Austria) and ImageJ software, respectively. The antibodies used are listed in Table S1.

Histological analysis and immunohistochemistry (IHC) assay

Tumor tissues were dissected and fixed with 4% paraformaldehyde. Subsequently, the paraffin sections were subjected to hematoxylin and eosin (HE) or IHC staining according to the following procedures. Paraffin embedding, sectioning and staining were performed by Zuocheng Company (Shanghai, China). For HE staining, sections were counterstained with hematoxylin. For IHC staining, after antigen retrieval by boiling in citrate buffer (10 mM sodium citrate buffer, pH 6.0) for 10 min and blocking, the slides were incubated with an anti-c-Myc antibody (ab32072, Abcam, Cambridge, UK) at 4°C overnight, followed by incubation with HRP-conjugated secondary antibodies at room temperature for 1 h, after which peroxidase activity was detected with diaminobenzidine (DAB, DAKO, Denmark). The images were acquired and processed using the Tissue FAXS Plus Basic. The mean densitometry of the digital image was used to determine the protein IHC score. The signal density of the tissue areas from five randomly selected fields was measured in a blinded manner and subjected to statistical analysis.

Enzyme linked immunosorbent assay (ELISA)

Tumor samples were homogenized in 50 mM Tris-HCl pH 7.5 containing 2 mM EDTA, 1% Triton X-100, 1 mM PMSF and protease inhibitors (Complete® -EDTA-free, Roche) (1 g tumor tissue/ml). Tissue homogenates were centrifuged at 4000 rpm for 30 minutes at 4°C, and the levels of IL-10 (Raybiotech, Georgia, USA), TGF-β (Raybiotech), and IL-8 (Abcam) in the supernatants were measured according to the manufacturer's instructions. Total protein concentrations were measured with a BCA Protein Assay Kit (Thermo Fisher Scientific).

Xenograft mouse model

Female nude mice (BALB/c nude, 5-6 weeks old) were purchased from Charles River Laboratories (Beijing, China) and used for the establishment of PDAC xenografts. All in vivo experiments were conducted under the institutional ethical guidelines on animal care and were approved by the Institute of Animal Care and Use Committee at the Shanghai Institute of Materia Medica, Chinese Academy of Sciences and Lingang Laboratory. Animal maintenance was performed in accordance with the guidelines of the Animal Experiment Center of Shanghai Institute

of Materia Medica and Lingang Laboratory. All mice were housed in a controlled environment with air filtration, temperature regulation (22-24°C), light management, humidity maintenance (40%-70%), and free access to a standard diet. Subsequently, single-cell suspensions were subcutaneously implanted into the right flank of the mice.

The mice were randomly allocated to either the control or treatment groups (with five mice per group), and dosing commenced once the average tumor volume reached approximately 100 mm^3. AZD4573 (5% DMSO+95% saline with 20% SBE-β-CD, i.p.), trametinib (5% DMSO+95% saline with 0.5% CMC-Na, p.o.), BI-3406 (5% DMSO+95% saline with 0.5% CMC-Na, p.o.), AMG510 (5% DMSO+5% PEG400 +90% saline with 0.5% CMC-Na, p.o.), MRTX849 (90% saline with 0.5% CMCNa + 5% PEG400 +5% DMSO, p.o.), and SB290157 (5% DMSO+95% saline with 20% SBE-β-CD, i.p.) were prepared at the indicated doses. Mice were treated for 3-4 weeks as indicated and tumor volumes were measured every two or three days by a microcaliper and calculated by the following formula: volume (mm^3) = ½ × length × $width^2$. Mouse body weight was measured every two or three days. Mice were euthanized and tumor tissues were collected 2 h after the last dosing for subsequent immunoblotting, IHC or IF staining and cytokine measurements. No blinding was implemented during animal experiments.

Statistical analysis

Statistical differences were determined using the two-sided unpaired Student's *t* test for two groups or one-way ANOVA followed by Tukey's multiple group comparison test for more than two groups. (GraphPad Prism 8.0). Survival curves were plotted according to the Kaplan–Meier method. The correlations were analyzed by Pearson's correlation coefficient (R). RNA-seq and IP-LC-MS/MS were repeated three and two times (biological replicates), respectively. Other experiments were performed at least three times (independent replicates with at least three biological replicates each). All data are reported as means ± standard deviations (SD) or standard error of the mean (SEM). The detailed n values for each panel in the figures are provided in the corresponding legends. Statistical significance was considered at $p<0.05$ (ns = not significant, *$p<0.05$, **$p<0.01$, and ***$p<0.001$).

References

[1] J. Schindelin, I. Arganda-Carreras, E. Frise, V. Kaynig, M. Longair, T. Pietzsch, S. Preibisch, C. Rueden, S. Saalfeld, B. Schmid, J. Y. Tinevez, D. J. White, V. Hartenstein, K. Eliceiri, P. Tomancak, A. Cardona, *Nature methods* **2012**, *9* (7), 676, https://doi.org/10.1038/nmeth.2019.

[2] a) V. Vichai, K. Kirtikara, *Nature protocols* **2006**, *1* (3), 1112, https://doi.org/10.1038/nprot.2006.179; b) J. W. Taub, X. Huang, L. H. Matherly, M. L. Stout, S. A. Buck, G. V. Massey, D. L. Becton, M. N. Chang, H. J. Weinstein, Y. Ravindranath, *Blood* **1999**, *94* (4), 1393.

[3] T. T. Chang, T. C. Chou, *Acta paediatrica Taiwanica = Taiwan er ke yi xue hui za zhi* **2000**, *41* (6), 294.

[4] K. Dittmann, C. Mayer, B. Fehrenbacher, M. Schaller, U. Raju, L. Milas, D. J. Chen, R. Kehlbach, H. P. Rodemann, *The Journal of biological chemistry* **2005**, *280* (35), 31182, https://doi.org/10.1074/jbc.M506591200.
